# The *NOTCH1*/*SNAIL1/MEF2C* Pathway Regulates Growth and Self-Renewal in Embryonal Rhabdomyosarcoma

**DOI:** 10.1016/j.celrep.2017.05.061

**Published:** 2017-06-13

**Authors:** Myron S. Ignatius, Madeline N. Hayes, Riadh Lobbardi, Eleanor Y. Chen, Karin M. McCarthy, Prethish Sreenivas, Zainab Motala, Adam D. Durbin, Aleksey Molodtsov, Sophia Reeder, Alexander Jin, Sivasish Sindiri, Brian C. Beleyea, Deepak Bhere, Matthew S. Alexander, Khalid Shah, Charles Keller, Corinne M. Linardic, Petur G. Nielsen, David Malkin, Javed Khan, David M. Langenau

**Affiliations:** 1 Department of Pathology, Massachusetts General Hospital, Boston, MA 02114, USA; 2 Center of Cancer Research, Massachusetts General Hospital, Charlestown, MA 02129, USA; 3 Harvard Stem Cell Institute, Boston, MA 02114, USA; 4 Greehey Children's Cancer Research Institute and Department of Molecular Medicine, UT Health Sciences Center, San Antonio, TX 78229, USA; 5 Department of Pathology, University of Washington, Seattle, WA 98195, USA; 6 Division of Hematology/Oncology, Hospital for Sick Children and Department of Pediatrics, University of Toronto, Toronto, ON M5G 1X8, Canada; 7 Department of Pediatric Oncology, Dana-Farber Cancer Institute, Harvard Medical School, Boston, MA 02215, USA; 8 Division of Pediatric Hematology/Oncology, Boston Children's Hospital, Boston, MA 02215, USA; 9 Oncogenomics Section, Genetics Branch, Center for Cancer Research, National Cancer Institute, NIH, Bethesda, MD 20892, USA; 10 Department of Pediatrics and Department of Pharmacology and Cancer Biology, Duke University Medical Center, Durham, NC 27710, USA; 11 Molecular Neurotherapy and Imaging Laboratory, Stem Cell Therapeutics and Imaging Program, Massachusetts General Hospital, Harvard Medical School, Boston, MA 02114, USA; 12 Department of Pediatrics and Genetics, Children's of Alabama and the University of Alabama at Birmingham, Birmingham, AL 35233, USA; 13 Children's Cancer Therapy Development Institute, Beaverton, OR 97005, USA

## Abstract

Tumor-propagating cells (TPCs) share self-renewal properties with normal stem cells and drive continued tumor growth. However, mechanisms regulating TPC self-renewal are largely unknown, especially in embryonal rhabdomyosarcoma (ERMS)—a common pediatric cancer of muscle. Here, we used a zebrafish transgenic model of ERMS to identify a role for intracellular NOTCH1 (ICN1) in increasing TPCs by 23-fold. ICN1 expanded TPCs by enabling the de-differentiation of zebrafish ERMS cells into self-renewing myf5+ TPCs, breaking the rigid differentiation hierarchies reported in normal muscle. ICN1 also had conserved roles in regulating human ERMS self-renewal and growth. Mechanistically, ICN1 up-regulated expression of *SNAIL1*, a transcriptional repressor, to increase TPC number in human ERMS and to block muscle differentiation through suppressing *MEF2C*, a myogenic differentiation transcription factor. Our data implicate the *NOTCH1/SNAI1/MEF2C* signaling axis as a major determinant of TPC self-renewal and differentiation in ERMS, raising hope of therapeutically targeting this pathway in the future.

## Graphical abstract

Tumor-propagating cells (TPCs) drive cancer growth, yet mechanisms regulating TPC self-renewal and maintenance are largely unknown. Ignatius et al. show that the NOTCH1/SNAIL1 pathway synergizes with RAS to expand TPCs in embryonal rhabdomyosarcoma. This pathway blocks *MEF2C*-induced differentiation and enables the de-differentiation of ERMS cells into self-renewing TPCs.

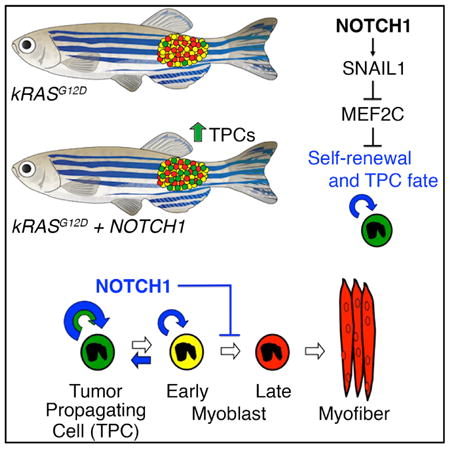

## Introduction

Self-renewal is the process by which cells make more of themselves and has been ascribed to both normal and malignant stem cell populations (Morrison and Spradling, 2008; Reya et al., 2001). In many cancers, tumor cells are hierarchically organized, with a distinct population of undifferentiated cells exclusively retaining self-renewal and long-term growth potential—these cells are commonly called tumor-propagating cells (TPCs). TPCs are often refractory to conventional chemotherapies, thereby leading to relapse and metastasis (Beck and Blanpain, 2013). While molecularly defined TPCs have now been identified in many cancers (Beck and Blanpain, 2013; Ignatius et al., 2012; Walter et al., 2011), the molecular pathways that drive cancer stemness, self-renewal, and differentiation arrest are not well understood.

Rhabdomyosarcoma (RMS) is a common pediatric malignancy that shares morphologic and molecular features with embryonic skeletal muscle (Xia et al., 2002). RAS is a major oncogenic driver of the embryonal rhabdomyosarcoma (ERMS) subtype, with >90% of patients having activation of this pathway (Chen et al., 2013; Langenau et al., 2007; Shern et al., 2014). However, additional pathways are likely required for eliciting full transformation and imparting self-renewal to transformed cells. To date, only a single paper in the literature has defined acquired oncogenic pathways in regulating stem cell pathways in human ERMS, defining important roles for the Hedgehog signaling pathway in regulating TPC number in ERMS (Satheesha et al., 2016). However, despite this pathway being turned on in a fraction of human ERMS, activating mutations in the Hedgehog pathway are uncommon, and the extent to which this pathway contributes to ERMS to drive continued tumor growth by modulating TPC number is under active investigation. As with other tumors that sustain long-term growth through TPCs, it is likely that additional pathways regulate TPC number, growth, and maintenance in human rhabdomyosarcoma.

Zebrafish have become an important animal model to uncover evolutionarily conserved pathways that regulate rhabdomyosarcoma growth, TPC cell number, and self-renewal (Chen et al., 2014; Ignatius et al., 2012; Langenau et al., 2007; reviewed in Kashi et al., 2015). Using transgenic approaches, we have generated zebrafish that develop embryonal rhabdomyosarcoma that are molecularly and histopathologically similar to human disease (Langenau et al., 2007). The zebrafish model has also been used to assess tumor cell heterogeneity and to identify functions of molecularly defined cell fractions in driving tumor growth. For example, zebrafish can be created that express fluorescent proteins under control of muscle-specific promoters that are active only during specific states of muscle cell maturation (Ignatius et al., 2012). Using this approach, we have uncovered that *myf5:GFP+/mylz2-mCherry-negative* TPCs share similar characteristics with normal muscle satellite cells, can sustain tumor growth, and are regulated by similar molecular pathways (Chen et al., 2014; Ignatius et al., 2012; Langenau et al., 2007; reviewed in Kashi et al., 2015).

Building on our knowledge that muscle development, regeneration, and stem cell self-renewal are regulated by the NOTCH1 pathway (Conboy et al., 2003; Kuang et al., 2008), we undertook experiments to assess a role for NOTCH1 in regulating human rhabdomyosarcoma growth through specifically affecting TPCs. Our work uncovered important roles for intracellular NOTCH1 (ICN1) signaling in regulating self-renewal, differentiation arrest, and growth in zebrafish, mouse xenografts, and human cell culture. Functional studies showed that SNAIL1 *(SNAI1)* is activated downstream of *ICN1* in human ERMS and stimulated self-renewal and growth, in part, by repressing expression of the muscle differentiation transcription factor *MEF2C*. Our data provide a mechanism by which oncogenic NOTCH1 regulates the overall number and properties of tumor-sustaining cell types in ERMS and provides therapeutic targets for this disease.

## Results

### The Notch1 Pathway Expands the Number of TPCs in Zebrafish ERMS

**Figure 1:**
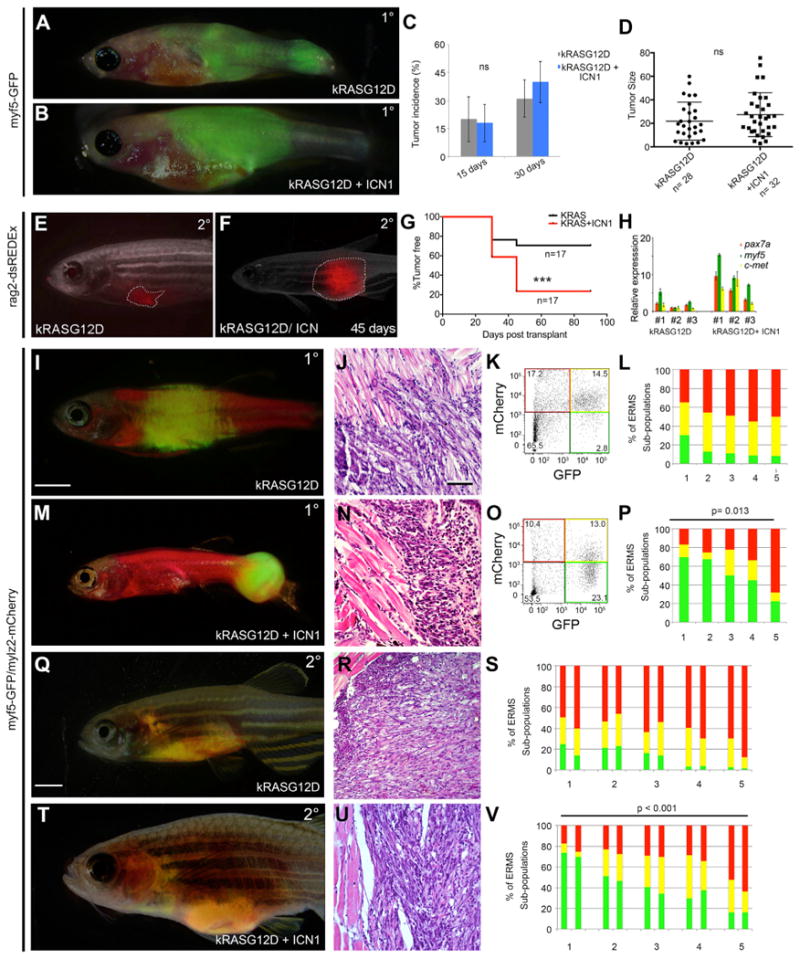
Notch1 Pathway Activation Increases the Number of myf5+ Progenitor Cells in Zebrafish ERMS (A and B) ERMS generated in *Tg(myf5-GFP)* zebrafish expressing (A) *kRASG12D* or (B) *kRASG12D + ICN1*. (C and D) Shown here: (C) tumor incidence and (D) size comparing ERMS at 15 and 30 days of life. Relative tumor size was measured at day 30. ns, not significant. (E and F) Images showing the difference in size of syngeneic zebrafish engrafted with 1 × 10^4^ bulk tumor cells labeled with *rag2-dsRedExpress* and imaged at day 45. Tumor boundaries are denoted by dashed lines. (G) Kaplan-Meijer analysis denoting differences in engraftment rates; n = 17 transplant animals per group from four independent tumors per group (p < 0.0001, log-rank statistic). (H) Real-time qPCR gene expression performed on sorted dsRedExpress+ ERMS cells arising within individual tumors. *p < 0.05, Student's t test. (I–P) Primary ERMS arising in *Tg(myf5-GFP; mylz2-mCherry*) animals. ERMS expressing (I–L) *kRASG12D* alone and (M–P) *kRASG12D + ICN1*. (I and M) Whole animal images, (J and N) H&E-stained sections, and (K, O, L, and P) representative flow cytometry. Graphical analysis showing percentages of fluorescent-labeled ERMS subpopulations within individual tumors following FACS. Five independent primary tumors were assessed, and each is denoted by numbers on the x axis. (p = 0.013, Student's t test). (Q–V) Transplanted ERMS arising from *Tg(myf5-GFP; mylz2-mCherry*) tumors. (Q–S) ERMS expressing *kRASG12D* alone and (T–V) *kRASG12D + ICN1*. (Q and T) Whole animal images of transplant animals, (R and U) H&E-stained sections, and (S and V) bar graphs showing fluorescent-labeled ERMS subpopulations following FACS. Five independent primary transplanted tumors were engrafted into CG1 fish, and each are denoted by numbers on the x axis. FACS populations for two representative engrafted fish per tumor are shown (p < 0.001, Student's t test). Scale bars in (I) and (Q), also pertaining to (M) and (T), 2 mm; scale bar in (J), also pertaining to (N), (R), and (U), 50 μm. See also Figure S1.

To assess a role for *NOTCH1* signaling in ERMS, we compared zebrafish ERMS cells that express *kRASG12D* with those that co-express both *kRASG12D* and *intracellular-activated NOTCH1* (*ICN1*)(Figures 1A and 1B). Gene expression analysis confirmed that transgenic *ICN1* was expressed at physiological levels found in normal development (Figure S1A). Primary ERMS onset, penetrance, and tumor size did not differ between tumors arising in *kRASG12D* or *kRASG12D+ICN1*-expressing fish (Figures 1C and 1D). However, following the cell transplantation of equal numbers of ERMS cells into syngeneic CG1 recipients (1 × 10^4^ cells per fish), fluorescent-labeled *ICN1-*expressing tumors grew qualitatively faster and had increased penetrance of disease (Figures 1E–1G; p = 0.012, log-rank/Mantel-Cox test). *ICN1*-expressing ERMS also expressed significantly higher levels of muscle stem cell genes, including *myf5*, *pax7a*, and *c-met* (Figure 1H). This gene signature is highly and specifically expressed in the *myf5-GFP+* ERMS TPCs (Ignatius et al., 2012). *ICN1*-expressing ERMS also exhibited a 23-fold increase in TPCs when assessed by bulk limit dilution cell transplantation into syngeneic recipient animals (Table S1; n = 4 tumors per group; p < 0.000001, extreme limiting dilution analysis [ELDA]). Our results have uncovered a dominant role for *NOTCH1* signaling in elevating the TPC number in zebrafish *kRASG12D*-induced ERMS.

**Figure 2:**
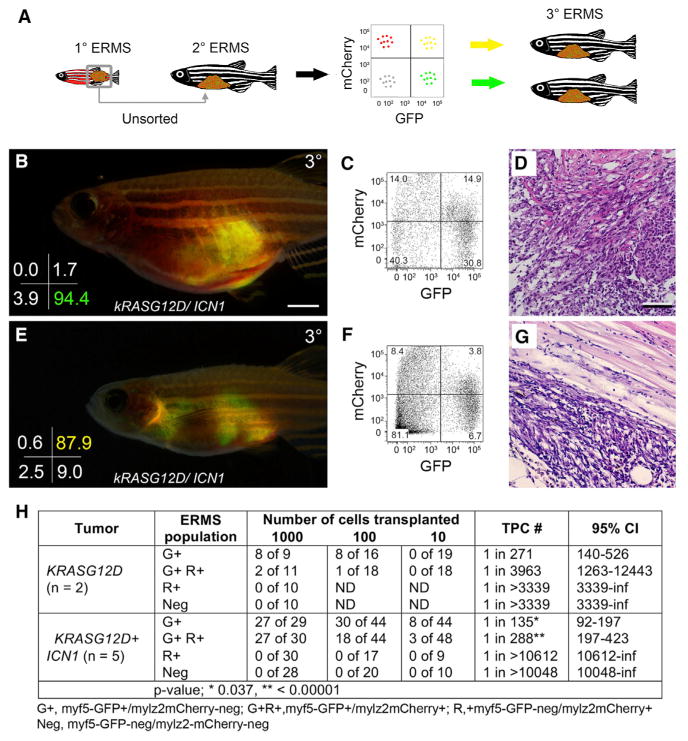
Notch1 Pathway Activation Confers Tumor-Propagating Activity to Differentiated ERMS Cells (A) Schematic of limiting dilution cell transplantation assay used to assess engraftment potential of fluorescently labeled ERMS cell fractions. (B–D) Engraftment with FACS-sorted *myf5-GFP+/mylz2-mCherry*-*negative* cells. (B) Whole animal image, (C) engrafted tumor cells analyzed by FACS, and (D) histology. Sort purity is denoted in the lower left corner of (B). (E–G) Engraftment with FACS-sorted double-positive *myf5-GFP+/mylz2-mCherry+* differentiated cells. (E) Whole animal image, (F) engrafted tumor cells analyzed by FACS, and (G) histology. Sort purity denoted in lower left corner of (E). (H) Table showing combined analysis of engraftment rates for *myf5-GFP+/mylz2-mCherry*-*negative*, double-positive *myf5-GFP+/mylz2-mCherry+*, *myf5-GFP-negative/mylz2-mCherry+*, and double-negative cells. Number of tumors analyzed per condition is noted. ND, not determined; CI, confidence interval; inf, infinity. Scale bar in (B), also pertaining to (E), 2 mm; scale bar in (D), also pertaining to (G), 50 μm. See also Figure S2, Table S2, and Table S3.

One explanation of our results is that *ICN1* may expand the number of previously defined *myf5-GFP+* TPCs (Ignatius et al., 2012). To directly test this hypothesis, we generated ERMS in syngeneic *myf5-GFP/mylz2-mCherry* transgenic fish. These fluorescent transgenic lines have been previously used to show that tumor-propagating activity is exclusively confined to the *myf5-GFP+/mylz2-negative* ERMS cells (Ignatius et al., 2012). Fluorescence-activated cell sorting (FACS) analysis revealed that primary *ICN1*-expressing ERMS had a 3-fold increase in the numbers of *myf5-GFP+/mylz2-negative* cells while also decreasing the more differentiated *mylz2-mcherry+* ERMS cells (Figures 1I–1P; n = 5 tumors per group; p = 0.013, Student's t test). Similar results were observed in ERMS that developed in transplant recipient fish (Figures 1Q–1V; n = 5 independent tumors per group; p < 0.001, Student's t test). Importantly, the *myf5-GFP+/mylz2-negative* ERMS cells continued to retain tumor-propagating activity when assessed by limiting dilution cell transplantation (Figure 2H; Table S2). Thus, ICN1 pathway activation expands the number of classically defined *myf5-GFP+/mylz2-negative* TPCs that have been previously shown to drive the growth of zebrafish *kRASG12D*-induced ERMS (Ignatius et al., 2012).

### *ICN1* Confers Tumor-Propagating Activity to Mid-differentiated ERMS Cells

*ICN1* increased molecularly defined *myf5-GFP+/mylz2-negative* TPCs 3-fold when compared with tumors that express only *kRASG12D*, yet *ICN1*-expressing ERMS cells had a 23-fold increase in TPCs when assessed by limiting dilution cell transplantation. These data suggested that *ICN1* could confer tumor-propagating ability to more differentiated ERMS cells. Mid-differentiated *myf5-GFP+/mylz2-mcherry+* ERMS from *ICN1*-expressing tumors expressed early muscle progenitor markers, including *myf5*, *c-met*, and *m-cadherin* yet retained more differentiated muscle gene expression, including *myoD*, *myogenin*, *mylz2*, *tnni2a*, *actc1b*,and *myh9a* (Figures S1B–S1N). We had previously shown that proliferation largely resided in the *myf5-GFP+/mylz2-mCherry*-*negative* ERMS population in *kRASG12D*-driven ERMS (Ignatius et al., 2012). By contrast, both the *myf5-GFP+/mylz2-mCherry*-*negative* and the mid-differentiated, double-positive cells from *ICN1*-expressing ERMS were highly proliferative when assessed for EDU incorporation following a 6-hr pulse (Figures S1S–S1U). These data suggest that mid-differentiated cells likely drive continued tumor growth in ICN1-expressing ERMS and that TPC function could be imparted to more differentiated ERMS cells.

To directly assess whether more differentiated ICN1-expressing ERMS cells had gained tumor-propagating potential, highly purified ERMS sub-populations were isolated by FACS and implanted into syngeneic recipient fish at limiting dilution (purity > 86.6%, and viability > 86.9%; Figure 2A). Consistent with previous findings (Ignatius et al., 2012), the *myf5-GFP+/mylz2-mCherry*-*negative* ERMS sub-population engrafted into recipient fish with no differences in engraftment frequencies between *kRASG12D* and *kRASG12D + ICN1*-expressing tumors (Figures 2B–2D, 2H, and S2A–S2E; Table S2; n = 5 tumors analyzed). By contrast, the mid-differentiated *myf5-GFP+/mylz2-mCherry+ ICN1-*expressing ERMS cells could also now robustly engraft a tumor (p < 0.01; Figures 2E–2H and S2F– S2J; Table S2). Serial transplantation experiments confirmed that both ICN1-expressing ERMS cell populations had long-term engraftment capacity (Figures S2F–S2P; Table S2).

One potential cellular mechanism by which mid-differentiated ERMS cells can drive tumor growth is to undergo de-differentiation and become classically defined *myf5-GFP+/mylz2-mcherry-negative* self-renewing TPCs. To test this possibility, we isolated highly purified mid-differentiated *myf5-GFP+/mylz2-mCherry+* ERMS cells (97.5% sort purity, >95% viable) and transplanted 10–20 cells into recipient fish. The calculated probability of en-grafting a tumor from a single TPC was calculated at >99.7% (Table S3). Sort purity was independently confirmed by confocal microscopy (n = 100 of 100 tumor cells were G+R+ [*myf5-GFP+/mylz2-mCherry+*]), obviating the possibility of contamination by classically defined *myf5-GFP+/mylz2-mCherry-negative* TPCs. Highly purified double-positive ERMS cells engrafted robustly and made ERMS tumors that contained all fluorescent tumor cell subfractions, including the less differentiated *myf5-GFP+/mylz2-mCherry-negative* ERMS cells (n = 3 of 3; Figures S2Q– S2V; Table S3). Taken together, we conclude that *ICN1* imparts tumor-propagating potential to mid-differentiated cells and enables these same cells to oscillate between cellular states, leading to the production of less differentiated ERMS cells that can self-renew and drive tumor growth.

### NOTCH1 Regulates Cell Growth, Self-Renewal, and Differentiation in Human ERMS

**Figure 3:**
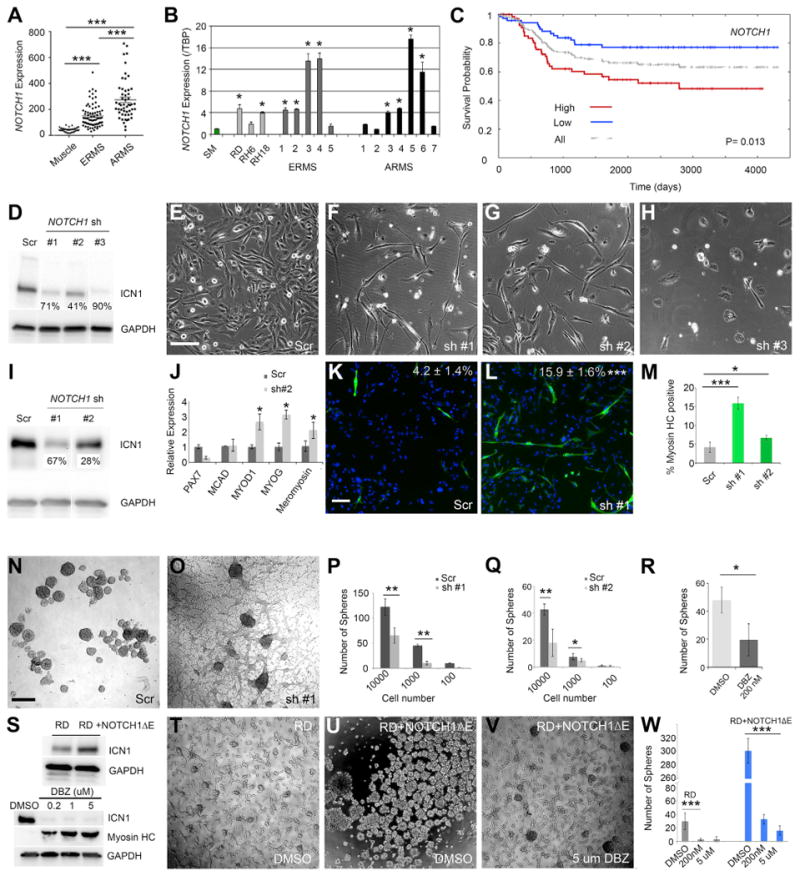
NOTCH1 Regulates Cell Growth, Differentiation, and Self-Renewal in Human ERMS (A) Microarray gene expression analysis of *NOTCH1* in human skeletal muscle and rhabdomyosarcoma. (B) qPCR gene expression of *NOTCH1* performed on skeletal muscle (SM), human rhabdomyosarcoma cell lines, and primary rhabdomyosarcoma. (C) Kaplan-Meijer analysis comparing survival in high versus low *NOTCH1* expression in rhabdomyosarcoma patients (p = 0.013, log-rank statistic, combined analysis of ARMS and ERMS, n = 128). (D) Western blot analysis of RD cells following control scrambled shRNA (Scr) or NOTCH1 knockdown using three independent lentiviral shRNA hairpins (sh). Percent knockdown is noted. (E–H) Morphology of RD cells after 5 days of shRNA treatment. (E) Control (Scr) and (F–H) NOTCH1 knockdown. (I) Western blot analysis showing ICN1 expression in stable RD knockdown cells. Percent knockdown is noted. (J) qPCR gene expression for a panel of muscle differentiation genes in control (Scr) and NOTCH1 knockdown RD cells. (K and L) Immunofluorescence staining for myosin heavy chain in stable RD cells expressing (K) scrambled or (L) NOTCH1 shRNA #1. Percentages of tumor cells with myosin heavy chain expression are denoted ± SD. (M) Quantitation of the percentage of tumor cells with myosin heavy chain expression in RD cells treated with control and two NOTCH1 shRNAs. (N–Q) Sphere formation in stable RD cells. Images of spheres from (N) scrambled or (O) NOTCH1 shRNA#1 knockdown cells. Quantitation of sphere colony formation when assessed at varying cell numbers for (P) NOTCH1 shRNA#1 or (Q) shRNA #2. (R) Sphere formation in human PDX PCB00234 ERMS cells treated with DMSO or Notch pathway inhibitor DBZ and assessed at 15 days. (S) Top: western blot analysis of RD cells with and without NOTCH1ΔE. Bottom: RD cells treated with DBZ for 10 days. (T–V) Sphere colony formation in RD cells treated with (T) DMSO and compared with RD cells expressing NOTCH1ΔE treated with (U) DMSO or (V) 5 μm DBZ. (W) Quantification of results. Scale bar in (E), also pertaining to (F)–(H), 200 μm; scale bar in (K), also pertaining to (L), 100 μm; scale bar in (N), also pertaining to (O), (T), and (V), 400 μm. Asterisks denote statistical differences by Student's t test (*p < 0.05; **p < 0.01; ***p < 0.001). Error bars represent ±1 SD. See also Figure S3.

**Figure 4:**
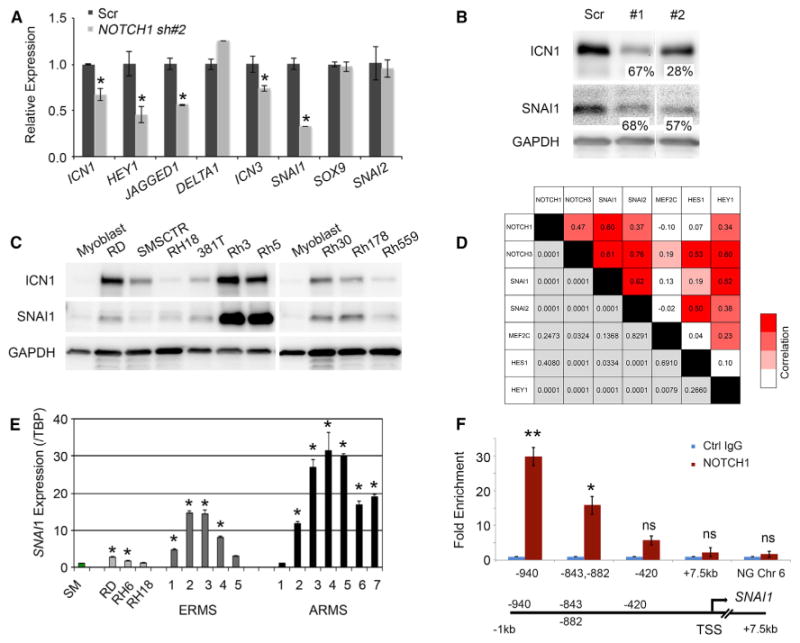
*SNAIL1* Is a Downstream Target of NOTCH1 in Human ERMS (A) qPCR gene expression of stable shRNA control (Scr) and NOTCH1 knockdown SMS-CTR cells. (B) Western blot analysis of RD cells following stable knockdown of NOTCH1. Percent knockdown is noted. ICN1 blot is the same as in Figure 3I. (C) Western blot analysis showing ICN1 and SNAI1 co-expression across human rhabdomyosarcoma cell lines. (D) Pearson correlation between the expression of *NOTCH1*, *NOTCH3*, *SNAI1*, *SNAI2*, *HES1*, *HEY1*, and *MEF2C* in primary human ERMS assessed by RNA-sequencing analysis. (E) qPCR gene expression of *SNAI1* performed on skeletal muscle (SM), human rhabdomyosarcoma cell lines, and primary rhabdomyosarcoma. TBP, TATA box-binding protein. (F) ChIP assay in RD ERMS cells followed by qPCR gene expression for NOTCH1-binding regions in a region 1 kb upstream of the *SNAI1* transcription start site (TSS). Ctrl, control; Chr, chromosome; NG, non geneic. Error bars in (A), (E), and (F) represent ±1 SD. In (A), *p < 0.05. In (E), *p < 0.01. In (F), *p < 0.05; **p < 0.01, Student's t test; ns, not significant. See also Figure S4.

To extend our findings to human ERMS, we first analyzed transcript expression of *NOTCH1* in primary patient tumors and uncovered that *NOTCH1* was highly expressed in 60% of both human alveolar rhabdomyosarcoma (ARMS) and ERMS when compared with normal muscle controls (Figure 3A). Importantly, transcript expression of well-known downstream targets of NOTCH1 was also concordantly deregulated, including *NOTCH3*, *HEY1*, and *JAGGED1* (Figures S3A–S3D). Real-time qPCR of primary human rhabdomyosarcoma independently confirmed that *NOTCH1* was highly expressed in a majority of tumors (n = 8 of 12 samples expressed a >4-fold increase in *NOTCH1* when compared with normal muscle; Figure 3B; Table S4). Next, ICN1 protein expression was assessed in a panel of human rhabdomyosarcoma cell lines, and we uncovered that all rhabdomyosarcoma cell lines expressed activated ICN1 to varying degrees (n = 9; Figure 4C). Finally, high *NOTCH1* expression was associated with reduced survival and poor outcome in human rhabdomyosarcoma patients (p = 0.013, log-rank statistic; Figure 3C). Rhabdomyosarcoma subtypes also exhibited a trend toward worse outcome based on high NOTCH1 expression (Figures S3E and S3F), yet these analyses failed to reach statistical significance due to small sample sizes. We conclude that *NOTCH1* signaling is found in a large fraction of human ERMS, likely identifying a group of high-risk patients.

To test the function of *ICN1* in human ERMS, we next performed knockdown of NOTCH1 using three different short hairpin RNAs (shRNAs) in RD and SMS-CTR cell lines, both of which are ERMS, have activating RAS mutations, and express high levels of ICN1 (Figures 3D–3H and S3G–S3J). All three shRNA constructs reduced ICN1 expression (Figures 3D and S3G). shRNA knockdown that reduced ICN1 expression by 90% led to loss of proliferation and cell death in RD lines (Figure 3H). By contrast, knockdown of ICN1 using two additional shRNAs resulted in 41%–71% reduction in ICN1 protein expression, yet it had significant effects on reducing growth (Figures 3F, 3G, and S3H–S3J). Following stable knockdown of ICN1 using these two shRNAs, both RD and SMS-CTR cells acquired morphological and gene expression changes associated with differentiation, while scrambled shRNA controls had no effect (Figures 3I–3M and S3K). These results are consistent with those reported previously by the C.M.L. group who identified roles for NOTCH1 in regulating differentiation arrest in human rhabdomyosarcoma (Belyea et al., 2011).

To test the role of ICN1 on regulating TPC number, we next used sphere formation assays under minimal growth factor conditions, a surrogate for assessing tumor-propagating activity in vivo (Walter et al., 2011). Knockdown of NOTCH1 with two independent shRNAs resulted in significantly reduced sphere formation in both RD and SMS-CTR cells (p < 0.01, Student's t test; Figures 3N–3Q and S3L–S3N). Furthermore, pharmacologic inhibition of NOTCH1 using the gamma-secretase inhibitor dibenzaze-pine (DBZ) reduced sphere formation in a human PDX ERMS (p < 0.05, Student's t test; Figure 3R). These results were independently confirmed in RD cells, where DBZ treatment reduced ICN1 protein expression (Figure 3S, bottom), enhanced differentiation as assessed by myosin heavy-chain (MF20) staining (Figures S3P–S3R), and reduced colony formation following 10 days of drug treatment (p < 0.001, Student's t test; Figure 3W). Conversely, gain-of-function experiments using constitutively active NOTCH1ΔE resulted in a 10-fold increase in sphere-forming ability in RD cells (Figures 3S, 3U, and 3W; p < 0.001, Student's t test). Because the membrane-bound NOTCH1ΔE requires cleavage by γ-secretase to become activated, we verified the specificity of our results by treating these cells with DBZ. DBZ treatment reduced sphere colony formation back to near-wild-type DMSO-treated control cells (p < 0.0001, Student's t test; Figures 3V and 3W). Our results establish a major role for the NOTCH1 signaling pathway in regulating TPC function and growth in human ERMS, consistent with our findings from the zebrafish model.

### *NOTCH1* Regulates *SNAI1* to Modulate TPC Function in Human ERMS

Gene expression studies were completed to identify potential downstream targets of *ICN1*. Not surprisingly, a subset of known *NOTCH1* target genes were reduced following shRNA knockdown of *ICN1* in both RD and SMS-CTR cell lines (*NOTCH3*, p = 0.001; *HEY1*, p = 0.02; and *JAGGED1*, p = 0.001, Student's t test; Figures 4A and S4A) (Belyea et al., 2011; Roma et al., 2011). *SNAI1*, *SNAI2*, and *SOX9* were also highly expressed in ERMS tumors and cell lines, compared to normal muscle; however, only *SNAI1* mRNA and protein expression were reproducibly reduced following shRNA knockdown in both RD and SMS-CTR cells (Figures 4A, 4B, S4A, and S4B). Transcriptional analysis of zebrafish ERMS had previously shown that *snai1a* and *snai2* were highly and specifically expressed in the TPC cell population in RAS-driven ERMS (Langenau et al., 2007). These same factors were also upregulated in ICN1-expressing ERMS and were highly expressed in both the *myf5-GFP+/mylz2-mCherry*-*negative* and mid-differentiated *myf5-GFPmylz2-mCherry+* subpopulations in ICN1+ ERMS (Figures S1Q and S1R) (Langenau et al., 2007). Since *SNAI1-*related family members have been implicated in regulating self-renewal in both normal and malignant stem cells in the breast epithelium (Mani et al., 2008; Ye et al., 2015), we reasoned that these factors might be downstream targets of ICN1 and regulate self-renewal in human ERMS.

Remarkably, rhabdomyosarcoma cell lines expressing high ICN1 also co-expressed high SNAI1 protein, correlating well with graded gene expression between the two factors (Figure 4C). Gene expression analysis also uncovered a high correlation of *SNAI1* and *NOTCH1* transcript co-expression in human primary rhabdomyosarcoma (Figure 4D; p < 0.0001; Pearson correlation, 0.604). This correlation was even higher than well-known *NOTCH1* target genes *NOTCH3* and *HEY1* (Figure 4D). Additional gene expression studies confirmed that high-*NOTCH1*-expressing primary tumors also had high *SNAI1* (Figure 4E; n = 8 of 8). Finally, chromatin immunoprecipitation (ChIP) experiments showed that *SNAI1* was a direct transcriptional target of NOTCH1, with NOTCH1 binding enriched >15-fold within a 1-kb region upstream of the *SNAI1* promoter (Figure 4F; p < 0.01).

We also assessed whether *NOTCH1* signaling was correlated with Hedgehog and canonical WNT/b-catenin signaling pathways previously shown to be associated with stemness and differentiation in rhabdomyosarcoma (Chen et al., 2014; Satheesha et al., 2016; Zibat et al., 2010). We found that *NOTCH1* transcript expression highly correlated with *GLI3* and, to a lesser extent, with *GLI1*; however, *NOTCH1* expression did not correlate with either *PTCH1* or *PTCH2*, or with Hedgehog-associated embryonic stem cell gene *NANOG*, suggesting that Notch and Hedgehog programs may regulate distinct self-renewal programs in rhabdomyosarcoma (Figures S4D and S4E). Similarly, there was no correlation between *NOTCH1* and canonical WNT target genes *AXIN2* and *DKK1* or stem cell genes previously identified to drive TPC growth in human rhabdomyosarcoma, including *NANOG*, *POU5F1*,or *SOX2*. Together, these results show that *SNAI1* is a bona fide downstream target of *NOTCH1* in ERMS and suggest non-overlapping roles for the *NOTCH1/SNAI1* axis in regulating stem cell programs that drive tumor growth.

**Figure 5:**
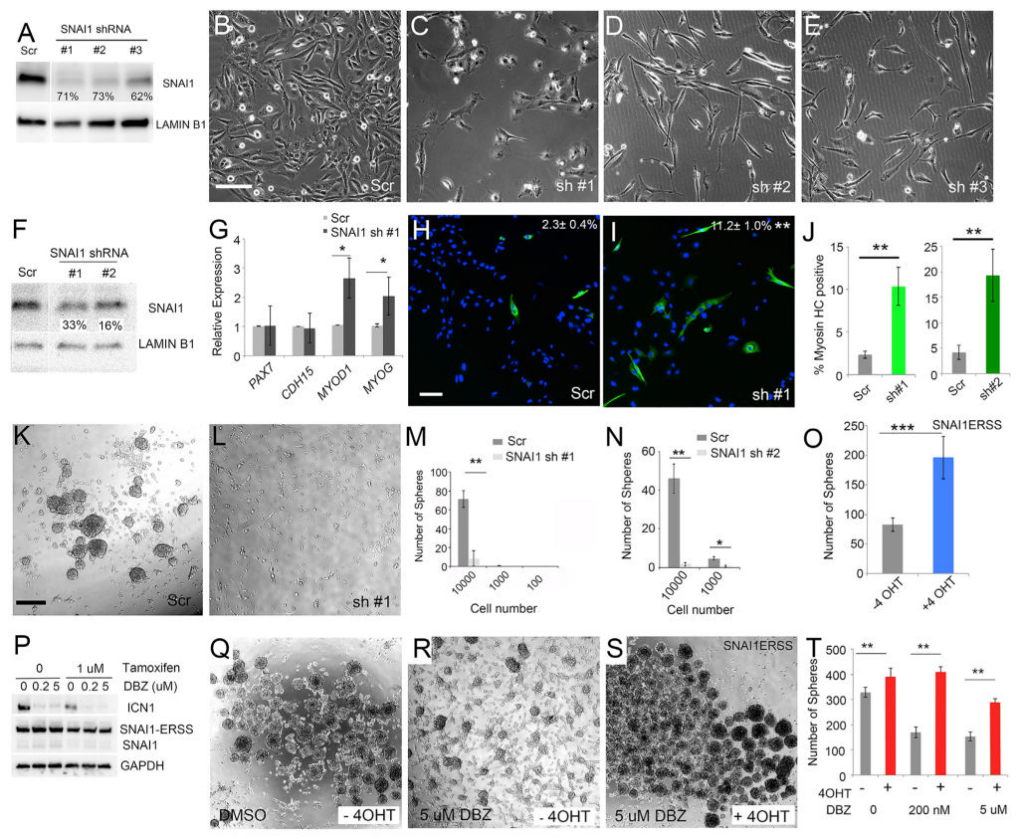
*SNAI1* Regulates Cell Growth, Differentiation, and Self-Renewal in Human ERMS (A) Western blot analysis of RD cells following control shRNA (Scr) or *SNAI1* knockdown using three independent lentiviral shRNA hairpins. Percent knockdown is noted. (B–E) Morphology of RD cells after 5 days post of shRNA treatment. (B) Control (Scr) and (C–E) *SNAI1* knockdown. (F) Western blot analysis showing SNAI1 expression in stable RD knockdown cells. (G) qPCR gene expression for a panel of muscle differentiation genes in RD knockdown cells. (H and I) Immunofluorescence staining for myosin heavy chain in stable RD cells expressing (H) control or (I) *SNAI1* shRNA. Percentage of tumor cells with myosin heavy chain expression are denoted ± SD. (J) Quantitation of the percentage of tumor cells with myosin heavy chain expression in RD cells treated with control and *SNAI1* shRNAs. (K–N) Sphere formation in stable RD cells. Images of spheres from (K) scrambled or (L) *SNAI1* shRNA#1 knockdown cells. Quantitation of sphere colony formation when assessed at varying cell numbers for control shRNA, (M) *SNAI1* shRNA #1, or (N) *shRNA#2*. (O) Sphere formation in RD cells stably expressing SNAI1-ERSS with and without 4-hydroxytamoxifen (4 OHT) treatment. (P) Western blot analysis of RD cells that stably express SNAI1-ERSS. Cells were treated for 10 days with DBZ and/or tamoxifen as noted. (Q–S) Sphere formation in RD cells expressing SNAI1-ERSS and treated for 10 days with (Q) DMSO, (R) DBZ, or (S) DBZ and tamoxifen as noted. (T) Quantification of data shown in (Q)–(S). Scale bar in (B), also pertaining to (C)–(E), 200 μm; scale bar in (H), also pertaining to (I), 100 μm; scale bar in (K), also pertaining to (L), (Q), (R), and (S), 400 μm. Asterisks denote significant differences based on Student's t test (*p < 0.05; **p < 0.01; ***p < 0.001). Error bars indicate ±1 SD. See also Figures S4 and S5.

We next assessed roles for *SNAI1* in regulating ERMS differentiation and self-renewal using shRNA knockdown in both RD and SMS-CTR cells. Similar to NOTCH1 knockdown, SNAI1 loss also resulted in reductions in cell number and acquisition of morphological characteristics associated with differentiation (three independent shRNAs; Figures 5A–5E and S5A–S5E). Stable knockdown moderately decreased SNAI1 protein expression (Figure 5F) yet had profound effects on increasing differentiation and reducing ERMS self-renewal in sphere colony formation assays performed in RD cells (p < 0.01, Student's t test; Figures 5F–5N). Consistent with *SNAI1* regulating self-renewal, expressing *SNAI1* using tamoxifen-inducible *SNAI1* (SNAI1ERSS) (Javaid et al., 2013) resulted in enhanced sphere formation (p < 0.001, Student's t test; Figures 5O and S5F–S5H). Finally, epistasis experiments were completed to show that *SNAI1* is downstream of NOTCH1. For example, stable knockdown of *ICN1* led to increased differentiation in RD and SMS-CTR cells. Following the addition of 4-hydroxytamoxifen and activation of SNAI1ERSS activity in these cells, differentiation was blocked (Figure S5I). Similarly, reducing ICN1 levels with DBZ in RD cells resulted in reduced sphere colony formation, which was rescued by activation of the SNAI1ERSS construct (Figures 5P-5T and S5J; p < 0.001, Student's t test). These experiments show that *SNAI1* is activated downstream of *NOTCH1* and is required for regulating self-renewal and differentiation of human ERMS.

### *NOTCH1* and *SNAI1* Are Required for ERMS Xenograft Growth and Maintenance

**Figure 6:**
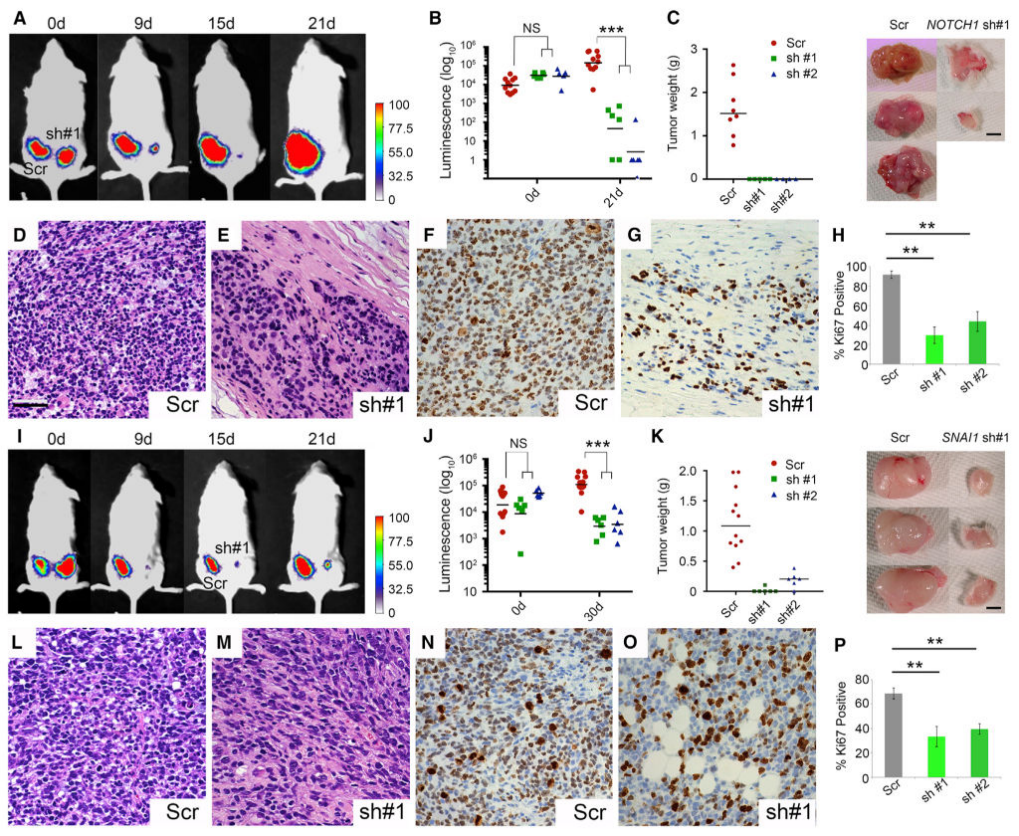
*NOTCH1* and *SNAI1* Are Required for Growth and Maintenance of Human ERMS following Xenograft Transplantation into Mice (A–H) *NOTCH1* knockdown suppresses RD growth in xenograft-transplanted mice. (A) Luciferase bioluminescent imaging of subcutaneously engrafted RD cells following stable shRNA knockdown and injection into the flanks of NOD/SCID/IL2g null mice. Scrambled (Scr; left) or NOTCH1 knockdown (right). Representative animal shown. d, days. (B) Quantitation of tumor growth. Error bars represent ±1 SD. (C) Quantitation of tumor weight following excision at necropsy (p < 0.0001, Fisher's exact test). Error bars are ± 1 SD. Representative tumors are shown at right. (D and E) Representative images of histology from engrafted tumors. (F–H) KI67 staining in (F) and (G) and quantification in (H). (I–P) *SNAI1* knockdown suppresses RD growth in xenograft-transplanted mice. (I) Luciferase bioluminescent imaging of engrafted mice. Scrambled (Scr; left) or *SNAI1* knockdown (right). (J) Quantitation of tumor growth. Error bars represent ± 1 SD. (K) Quantitation of tumor weight following excision at necropsy performed between 88 and 93 days post-transplantation (p < 0.0001, Fisher's exact test). Representative tumors are shown at right. Error bars are ± 1 SD. (L and M) Representative images of histology from engrafted tumors. (N–P) KI67 staining, in (N) and (O), and quantification, in (P), of the data shown in (N) and (O). Scale bar in (D), also pertaining to (E)–(G) and (L)–(O), 50 μm. Asterisks denote significant differences based on Student's t test (**p < 0.01; ***p < 0.001). NS, not significant. See also Figure S6.

Given that *NOTCH1* and *SNAI1* have potent effects on ERMS self-renewal in vitro, we expected that inactivation of either factor would result in reduced xenograft growth in mice. As expected, knockdown of either *NOTCH1* or *SNAI1* significantly impaired RD xenograft growth when compared with scrambled-control-infected cells (Figures 6A–6C, 6I–6K, S6A–S6E, and S6F–S6J; p < 0.001). By 30 days post-transplantation, 9 of 12 *NOTCH1* knockdown tumors had fully regressed, while all control tumors had grown substantially (Figures 6A and 6B). Similarly, knockdown of *SNAI1* in RD cells using two different shRNAs resulted in significantly reduced tumor growth in vivo when assessed by luciferase imaging at 21 days and after tumors were palpable (Figures 6I–6K and S6F–S6J; p < 0.001, two-way ANOVA followed by Dunnett's multiple comparisons test).

At necropsy, tumors isolated from *NOTCH1* and *SNAI1* knockdown xenografts weighed significantly less (Figures 6C and 6K; p < 0.001, Student's t test). Tumors were stained by H&E and revealed loss of cellularity in both *ICN1* and *SNAI1* knockdown tumors (Figures 6D, 6E, 6L, and 6M). Further, KI67 staining revealed that both *NOTCH1* and *SNAI1* knockdown tumors were significantly less proliferative when compared to scrambled-control-shRNA-expressing RD cells (Figures 6F–6H, 6N–6P; p < 0.002, Student's t test). Together, these data support roles for *ICN1* and *SNAI1* in regulating continued tumor growth and maintenance in vivo.

### *MEF2C* Is Repressed by *NOTCH1/SNAI1* Signaling and, when Activated, Leads to ERMS Differentiation and a Loss of Self-Renewal

**Figure 7:**
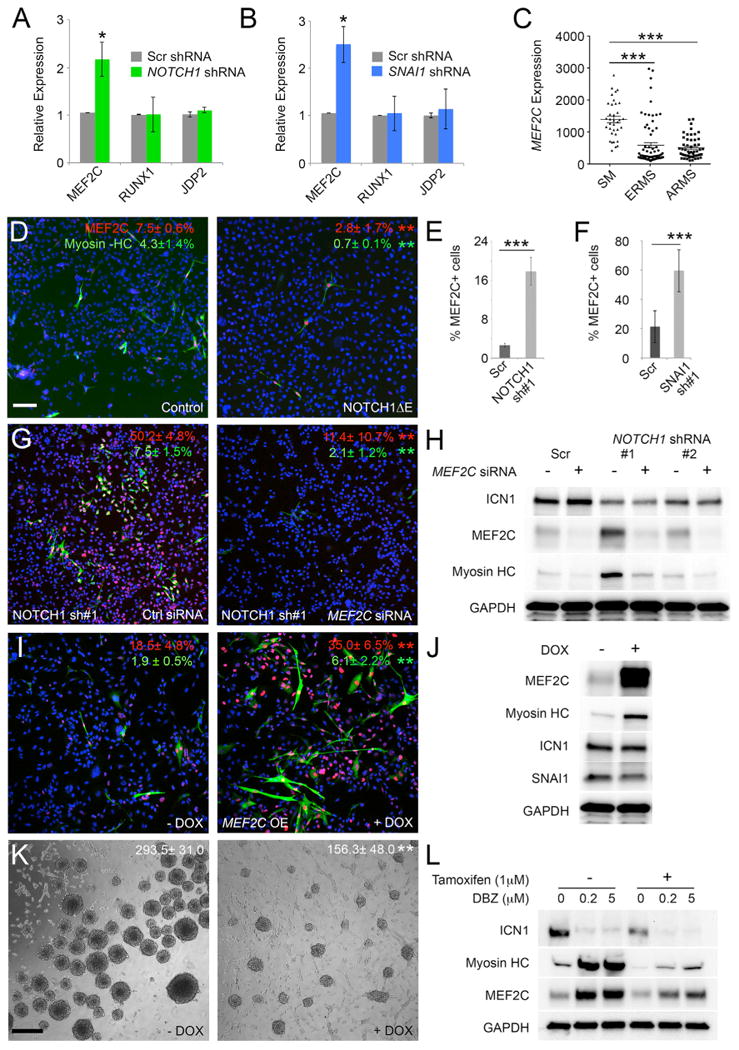
The *NOTCH1/SNAI1* Signaling Axis Suppresses MEF2C to Block Differentiation and to Elevate Human ERMS Self-Renewal (A) qPCR for *MEF2C*, *RUNX1*, and *JDP2* following *NOTCH1* knockdown in RD cells. Scr, scrambled. (B) qPCR expression following *SNAI1* knockdown in RD cells. (C) Microarray gene expression analysis of *MEF2C* in human skeletal muscle (SM) and rhabdomyosarcoma. (D) Immunofluorescence staining of RD- and RD+ NOTCH1ΔE-expressing cells. Red indicates MEF2C, green indicates myosin heavy chain, and blue indicates DAPI. (E) Quantification of the percentage of MEF2C-positive RD cells following stable knockdown with scrambled or *NOTCH1* shRNA. (F) Quantification of the percentage of MEF2C-positive RD cells following knockdown with *SNAI1* shRNA. (G) Immunofluorescence staining performed on NOTCH1 knockdown cells following treatment with control siRNA or *MEF2C* siRNA. (H) Western blot analysis of stable NOTCH1 knockdown cells following treatment with control siRNA or *MEF2C* siRNA. (I) Immunofluorescence staining following doxycycline-inducible MEF2C expression. -DOX, no doxycycline; +DOX, with doxycycline. OE, over expression. (J) Western blot analysis of human RD ERMS cells that have doxycycline-inducible expression of MEF2C. (K) Sphere formation in ERMS RD cells following doxycycline-inducible expression of MEF2C. Spheres assessed at 10 days of culture with colony number per 10,000 seeded cells are noted (±SD; p < 0.01). (L) Western blot analysis of RD cells that stably express SNAI1-ERSS cells. Cells were treated with 1 μM 4-hydroxytamoxifen to turn on SNAI1 activity and then were treated with DMSO or DBZ for 10 days in culture. Scale bar in (D), also pertaining to (G) and (I), 50 μm; scale bar in (K), 400 μm. Asterisks denote significant differences based on Student's t test: *p < 0.05; **p < 0.01; ***p < 0.001. See also Figure S7.

Finally, we explored the downstream molecular pathways deregulated by *NOTCH1/SNAI1* to uncover how this signaling axis regulates differentiation and self-renewal. MacQuarrie et al. (2013) have elegantly shown that promyogenic factors *JDP2*, *MEF2C*, and *RUNX1* are actively repressed in human ERMS RD cells and, when reactivated, can potently induce differentiation. Remarkably, expression of *RUNX1* and *JDP2* were unaffected by *NOTCH1 or SNAI1* knockdown, while only *MEF2C* was re-expressed (Figures 7A and 7B). Further, gene expression analysis showed that *MEF2C* was expressed at lower levels in both human ERMS and ARMS when compared to normal muscle (Figure 7C; p < 0.001, Student's t test). An immunohistochemistry (IHC) analysis confirmed that MEF2C was expressed in only a small fraction of human ERMS cells (Figure 7D), yet constitutively active NOTCH1ΔE could further reduce MEF2C+ cell numbers by 2.5-fold (p < 0.01, Student's t test; Figure 7D). Conversely, knockdown of either *NOTCH1 or SNAI1* resulted in increased numbers of MEF2C+ cells when assessed by IHC analysis (p < 0.001; Figures 7E and 7F).

To confirm that MEF2C is a downstream target of the *NOTCH1/SNAI1* signaling axis, epistasis experiments were completed. Knockdown of MEF2C in the setting of NOTCH1 silencing lead to reduced differentiation of ERMS cells (p < 0.01, Figures 7G and 7H). To test whether MEF2C was able to modulate differentiation and self-renewal of ERMS, we next engineered cells to express a doxycycline-inducible form of MEF2C. Following the overexpression of MEF2C, RD cells underwent terminal differentiation and expressed high levels of myosin heavy chain (Figure 7I and 7J). MEF2C also had important roles in modulating self-renewal in sphere colony formation assays where elevated MEF2C expression resulted in a 50% reduction in sphere colony formation (Figure 7K; p < 0.01, Student's t test). Finally, we performed additional epistasis experiments to show that SNAI1 is downstream of NOTCH1 and regulates MEF2C-induced differentiation (Figure 7L). RD cells expressing a tamoxifen-inducible SNAI1 construct were treated with or without DBZ to suppress NOTCH1 activity. As expected, DBZ reduced ICN1 levels concomitant with elevated differentiation and expression of both MEF2C and myosin heavy chain. Following the reactivation of SNAI1 by treating cells with tamoxifen, differentiation was severely impaired, with reduction in both MEF2C and myosin heavy chain protein expression, indicating that NOTCH1 signaling through SNAI1 leads to the block of ERMS differentiation (Figure 7L). Gene expression analysis of zebrafish ICN1-expressing ERMS also suggested the use of this molecular pathway in regulating differentiation and TPC self-renewal (Figure S7). In total, our experiments show that the *NOTCH1/SNAI1* axis suppresses *MEF2C* expression, locking cells in a less differentiated cell state while elevating the overall self-renewal potential of rhabdomyosarcoma.

## Discussion

Only a single report in the literature has assessed a role for ICN1 in regulating human ERMS growth in vivo, which was largely attributed to regulation of the downstream transcriptional activation of *HEY1* (Belyea et al., 2011). Despite these studies uncovering important roles for NOTCH1 in regulating rhabdomyosarcoma growth, roles for *NOTCH1* or *HEY1* in regulating stem cell programs that elevate overall numbers of TPCs was not reported. Rather, our work has uncovered prominent oncogenic roles for NOTCH1 in regulating the balance between ERMS self-renewal and differentiation, prominently impacting overall tumor growth. Remarkably, using the exceptional ability of our in vivo zebrafish ERMS model to differentially label ERMS tumor cells based on molecularly defined differentiation states, we also show that NOTCH1 pathway activation can break the rigid muscle stem cell hierarchies and modulate cell-state transitions between TPC and differentiated, non-proliferative cell populations. Importantly, these oscillating cell-state transitions are absent in ERMS expressing *kRAG12D* alone and differ markedly from the well-described roles for Notch1 in regulating symmetric stem cell divisions within the muscle satellite cell pool that increases the overall number of stem cells after injury (Conboy et al., 2003; Kuang et al., 2008).

Our in vivo findings that the *NOTCH1* pathway breaks rigid stem cell hierarchies and can modulate cell-state transitions in ERMS was unexpected. Plasticity of the stem cell fate is an uncommon phenomenon, with only a few reports published in normal and malignant contexts. For example, in vivo cell-lineage tracing of intestinal epithelial cells was used to show that differentiated cells can repopulate the stem cell niche only after injury by de-differentiating into stem cells (Buczacki et al., 2013). Similar reports have now been seen in the lung and the liver where NOTCH1 can directly reprogram differentiated cells into less differentiated progenitors that drive regeneration (Lafkas et al., 2015; Yimlamai et al., 2014). In melanoma, tumor cells can oscillate between stem-cell-like states by expressing JARIDB (Roesch et al., 2010), reconciling the lack of cell-surface markers that identify pure populations of TPCs and suggesting that extreme cell-state transitions and plasticity can be found in a subset of cancers (Quintana et al., 2008). To date, many of these studies lack plausible molecular mechanisms to account for these shifting cell fates. In ERMS, we show that the *NOTCH1/SNAI1/MEF2C* pathway regulates self-renewal and de-differentiation. Further, given the overall high conservation of molecular pathways used in normal and malignant muscle, we posit that ICN1 may have similar roles in de-differentiating myoblasts during injury into functional stem cells.

We have found that *NOTCH1* transcriptionally regulates *SNAI1*, a neural crest cell and EMT (epithelial-to-mesenchymal transition)-expressed factor whose expression and function in muscle is largely unexplored. Rather surprisingly, we find that *SNAI1* is highly expressed in rhabdomyosarcoma tumors and that knocking it down reduces self-renewal and growth while elevating tumor differentiation. Our understanding of *SNAI1* function is largely defined in neural crest cells, cancers of epithelial origin, and endothelial cells, where it is found to function as a transcriptional repressor that is also a part of chromatin-modifying complexes that regulate cell motility and EMT (Nieto et al., 2016; Timmerman et al., 2004). However, *SNAI1* and other EMT factors have also been recently implicated in regulating stemness in normal and malignant breast epithelial tissues. For example, Mani et al. (2008) explored the relationship between EMT factors and stemness in immortalized human mammary epithelial cells and discovered that the EMT program can reinitiate stem cell features, including the ability to form mammo-spheres and to increase efficiency of engraftment in vivo. Building on these findings, Guo et al. (2012) and Ye et al. (2015) found that an EMT network directed by *SNAI2* can regulate mammary stem cell properties in vitro and that co-expression of *SNAI2* and *SOX9* in human breast cancer cells enhanced tumorigenic ability and metastatic spread. In addition to regulating stemness and EMT-associated processes in epithelial tumors, our findings point to *SNAI1* function in regulating stemness in ERMS.

In ERMS, *SNAI1* modulates stem cell self-renewal programs by suppressing the expression of *MEF2C*, a well-known transcriptional activator that binds MYOD-binding sites and is required for robust terminal differentiation of myoblasts (MacQuarrie et al., 2013). Thus far, only a single report implicates a role for *SNAI1* in regulating *MYOD* transcriptional networks during muscle differentiation. In this report, Soleimani et al. (2012) found that SNAI1 preferentially binds at GC-rich E-box elements enriched in differentiating myotubes, thereby competing with MYOD for access to enhancers and promoters that regulate myoblast differentiation. Our work has uncovered unexpected roles for *MEF2C* in blocking self-renewal in addition to enhancing differentiation in human ERMS, suggesting that *MEF2C* acts as a molecular switch that regulates both self-renewal and differentiation programs downstream of *NOTCH1* and *SNAI1*. Indeed, others have shown that *MEF2C* is poorly expressed in human ERMS and, when re-expressed, leads to potent differentiation of RD cells (MacQuarrie et al., 2013). Thus, it appears that differentiation is not just the default state of rhabdomyosarcoma cells that exit the cell cycle. Rather, once differentiation programs are initiated by MEF2C, self-renewal programs are actively turned off, suggesting important insights into how self-renewal and differentiation programs are reciprocally regulated in muscle and rhabdomyosarcoma growth.

Our results are particularly important, as NOTCH1 inhibitory antibodies are now being assessed in preclinical models that have increased specificity and less gastrointestinal toxicity as pan-g-secretase inhibitors (Wu et al., 2010), raising hope that these strategies might be applied to the treatment of rhabdomyosarcoma in the future. Finally, given the importance of *NOTCH1* as an oncogene in other cancers (Ranganathan et al., 2011), it is likely that similar effects on self-renewal, stemness, and cell-state transitions will be observed in other Notch-driven tumors.

## Experimental Procedures

### Animals

Animal studies were approved by the Massachusetts General Hospital Subcommittee on Research Animal Care under protocols #2011-N-000127 (zebrafish) and #2013N000038 (mouse) and by the Partners Human Research Committee under institutional review board (IRB) protocol #2009-P-002756 (human).

Zebrafish used in this work include: CG1 strain, *myf5*-GFP transgenic zebrafish, *myf5-GFP/mylz2-mCherry* double-transgenic CG1-strain syngeneic zebrafish (Ignatius et al., 2012). NOD (non-obese diabetic)/SCID (severe combined immunodeficiency)/IL2g null mice used in this study were obtained from Jackson Laboratory.

### Micro-injection and ERMS Generation

The *rag2-kRASG12D*, *rag2-ICN1* (zebrafish intracellular notch1a), *rag2-GFP*, and *rag2-dsREDexpress* constructs were linearized with *Xho1*, phenol:chloroform extracted, ethanol precipitated, resuspended in 0.5 × Tris-EDTA + 0.1 M KCl, and injected into one-cell-stage embryos of the respective backgrounds, as previously described (Langenau et al., 2007).

### Quantification of Zebrafish Rhabdomyosarcoma Size and Initiation

Zebrafish were followed for tumor onset using an epifluorescent stereomicro-scope. Primary tumor size was quantified at 30 days of age using fluorescence intensity multiplied by the pixel area using the ImageJ software package as described previously (Chen et al., 2014). Kaplan-Meier tumor onset analysis was performed using GraphPad Prism software.

### FACS and ERMS Cell Transplantation

Zebrafish ERMS tumor cells were fluorescently labeled with GFP, dsRED, or mCherry and stained with DAPI to exclude dead cells and were sorted twice using a Laser BD FACSAria II Cell Sorter. Sort purity and viability were assessed after two rounds of sorting when possible, exceeding 85% and 95%, respectively. Sorted ERMS cells were transplanted into syngeneic CG1 fish and monitored for tumor engraftment under a fluorescent dissecting microscope from 10 to 120 days post-transplantation. TPC frequency was quantified using the Extreme Limiting Dilution Analysis software (http://bioinf.wehi. edu.au/software/elda/).

### Gene Expression Analysis

Real-time qPCR was completed using the Roche Lightcycler 480 machine. PCR primers and specific conditions are provided in Table S5 and Supplemental Experimental Procedures. RNA isolation and cDNA preparation were performed as previously described (Chen et al., 2014).

### Bioinformatic Analysis of Human Rhabdomyosarcoma Samples

Previously published transcriptome data from 65 ERMS samples (Shern et al., 2014) were processed using a standard Tuxedo pipeline (Trapnell et al., 2012). The resulting gene expression were log_2_ transformed and standardized (*Z* scored) using a set of 63 normal tissue samples. Using the “Hmisc” package in R, the Pearson correlation was determined for specific genes.

### Human Rhabdomyosarcoma Cell Lines and PDX Tumor

The human RD cell line was obtained from the ATCC; SMS-CTR, 381T, Rh3, Rh5, and Rh30 cell lines were obtained from Dr. Corinne Linardic; the Rh18 (fusion-negative) cell line was obtained from Dr. Peter Houghton; and RMS176 and RMS559 ERMS cells were obtained from Dr. Jonathan Fletcher. The ERMS PDX tumor PCB00234 was obtained from Dr. Charles Keller under IRB protocol #2009-P-002756 and Partners Institutional Biosafety Committee (PIBC) #2012B000024.

### Western Blot Analysis

Total cell lysates from human rhabdomyosarcoma cell lines and human myoblasts were obtained following lysis in 2% SDS lysis buffer supplemented with protease inhibitors (Santa Cruz Biotechnology). Western blot analysis used primary antibodies: rabbit a-NOTCH1 (Abcam), a-Cleaved NOTCH1 (Cell Signaling Technology), a-SNAI1 goat pAB (R&D Systems), a-Myosin Heavy Chain mouse mAb (monoclonal antibody) myosin heavy chain (a-MF20, R&D), MEF2C rabbit mAb (CST); and secondary antibodies: HRP (horseradish peroxidase) anti-rabbit (CST, 7074) or HRP anti-mouse (GE Healthcare, NA93IV). Membranes were developed using an ECL reagent (Western Lightning Plus ECL, PerkinElmer; or sensitive SuperSignal West Femto Maximum Sensitivity Substrate, Thermo Scientific). Membranes were striped, rinsed, and re-probed with the respective internal control rabbit a-Lamin B1 (Abcam) or rabbit a-GAPDH (CST).

### ChIP Assay

Chromatin from 5 × 10^6^ RD cells was isolated and fixed with 1% formaldehyde, sonicated, and processed according to the manufacturer's protocols (ChIP Assay Kit, Millipore). Immunoprecipitation was performed using 5 mg rabbit anti-NOTCH1 antibody (Abcam) or rabbit immunoglobulin G (IgG) and Pierce Protein A/G Agarose (Thermo Scientific). The immunoprecipitated DNA was subjected to real-time PCR with primers that target the SNAI1 promoter with negative controls in a region 7.5 kb upstream and an ORF (open reading frame)-free region in chromosome 6. All signals were normalized against input by the percentage input calculation method and normalized to IgG signal. Significance was calculated by Student's t test.

### Immunofluorescence Staining

Cells were fixed at 72 hr post-transfection in 4% paraformaldehyde (PFA)/PBS, permeabilized in 0.5% Triton X-100/PBS, and incubated with rabbit a-MEF2C (CST) and a-myosin heavy chain (R&D) in 2% goat serum/PBS. Secondary antibody detection used Alexa 488 goat anti-mouse and Alexa Fluor 594 goat anti-rabbit (Invitrogen). Cells were counterstained with DAPI (1:10,000) and imaged. Images were processed in ImageJ and Adobe Photoshop.

### Lentiviral, Retroviral, and siRNA Knockdown

Scrambled control shRNA and gene-specific shRNAs were delivered on the pLKO.1-background vector and packaged using 293T cells. Retroviral particles were made in Plat-A packaging cells using FuGENE6 (Promega). rhabdomyosarcoma cells were infected with viral particles for 24 hr at 37°C with 4 μg/mL of polybrene (EMD Millipore). Gene-specific smart-pool or control small interfering RNAs (siRNAs) (Dharmacon, GE Life Sciences) (1 pmol) were reverse-transfected into cells using Lipofectamine RNAiMAX Transfection Reagent (Life Technologies) in flat, clear-bottom 96-well plates.

### Mouse Xenografts, Bioluminescent Imaging, and Caliper Measurements

Luciferized RD cells were co-infected with shRNA lentivirus as outlined earlier. At 3 days post-infection, cells were collected, counted, and analyzed by flow cytometry to determine viability using DAPI. Equal numbers of viable cells were then embedded into Matrigel at a final concentration of 1 × 10^6^ cells per 100 μL and injected subcutaneously into anesthetized mice. Tumor growth was monitored weekly using bioluminescence imaging following injection using Luciferin at 75 mg/kg (15 mg/mL injected intraperitoneally). Comparison between groups was performed using a Student's t test. When palpable, tumors were measured using a caliper scale to measure the greatest diameter and length, which were then used to calculate tumor volume.

## Supplementary Material

s1

## Highlights

- NOTCH1 expands the number of tumor-propagating cells (TPCs) in zebrafish and human ERMS
- Notch1 drives the de-differentiation of zebrafish ERMS cells into self-renewing TPCs
- A NOTCH1/SNAI1 pathway drives self-renewal and blocks MEF2C regulated differentiation
- Self-renewal and differentiation pathways are linked and viable therapeutic targets
