# Supplementary material for: The NOTCH1/SNAIL1/MEF2C Pathway Regulates Growth and Self-Renewal in Embryonal Rhabdomyosarcoma
Source: Cell Rep. Author manuscript; Available in PMC 2017 Aug 19. (PMC5563075; doi:10.1016/j.celrep.2017.05.061)
Supplement: s1 [file NIHMS880402-supplement-s1.pdf]

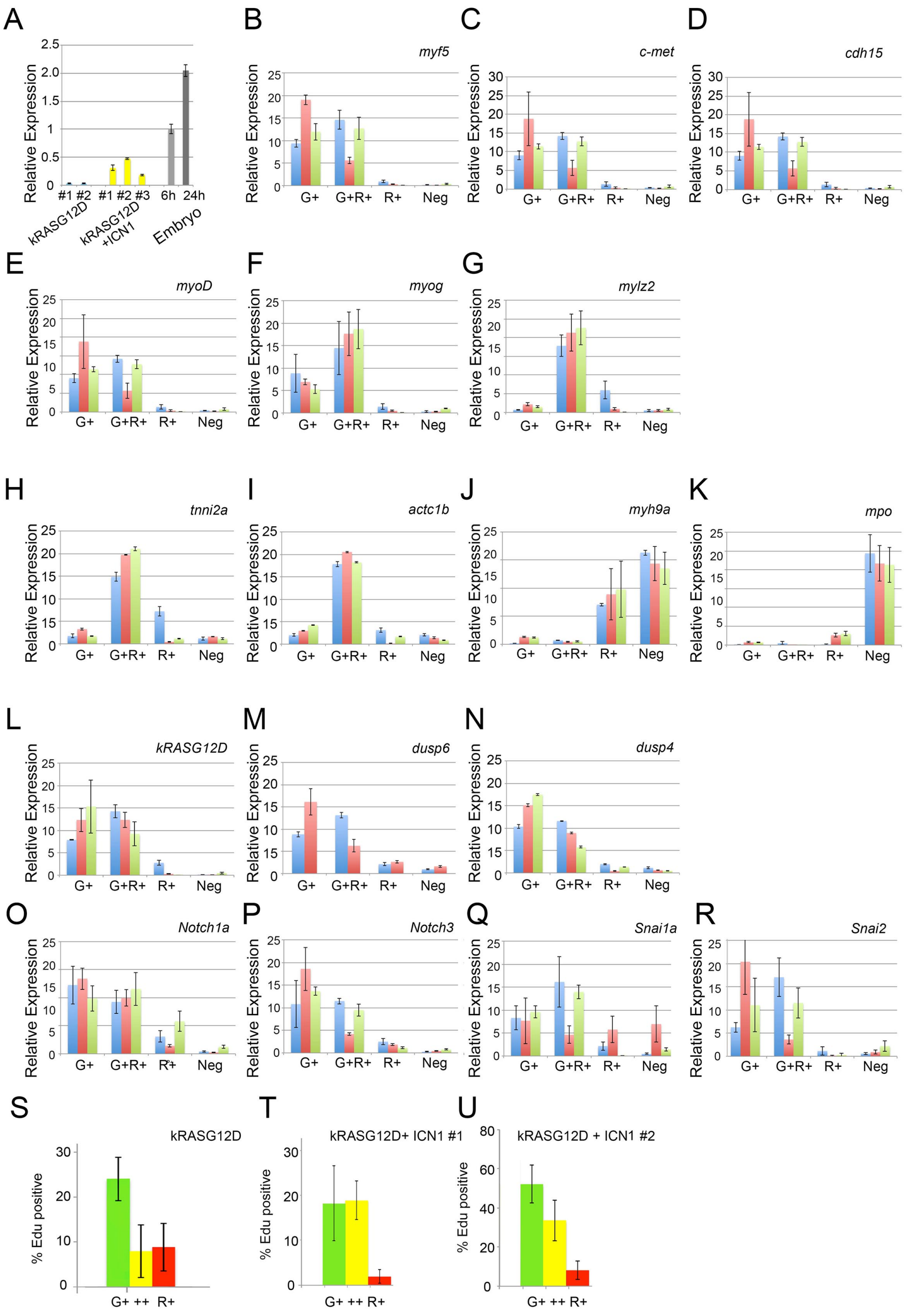

Figure S1. Notch1 pathway effects in transgenic zebrafish ERMS. Related to Figure 1

(A) Transgenic *ICN1* is expressed at physiologically relevant levels in zebrafish ERMS. Quantitative real-time PCR analysis of zebrafish ERMS that express *kRASG12D* or *kRASG12D+ ICN1* and compared with endogenous *NOTCH1* expression in 6 and 24 hour post-fertilization embryos. (B-R) Quantitative real-time PCR analysis of sorted ERMS cell subfractions isolated from three independent *kRASG12D + ICN1* expressing ERMS. *myf5-GFP+/mylz2-mCherry-negative* (G+), *myf5-GFP+/mylz2-mCherry+* (G+R+), *myf5-GFP-negative/mylz2-mCherry+*(R+), and double negative (Neg). *Myogenic regulatory factor (myf5), m-cadherin (cdh15), myogenic regulatory factor D (myoD), myogenin (myog), myosin light-chain 2 (mylz2), troponin I fast-twitch isoform 2 (tnni2a), alpha-actin 1b (acta1b), myosin heavy chain 9a (myh9a), myeloid-specific peroxidase (mpo), dual specificity phosphatase 4* and *6 (dusp4 and dusp6)*, *notch1a, notch3*, *snail1a (snai1a),* and *snail2 (snai2).* Expression is depicted as relative fold change where the total expression value of all transcripts was normalized to 25 within a given sample. The blue, red, and green bars represent analysis of three independent tumors. +/-1STD is denoted by error bars. (S-T) Both the *myf5-GFP+* and mid-differentiated *myf5-GFP+/mylz2-mCherry+* cells are highly proliferative in *ICN1*-expressing ERMS. EDU positivity in cell subfractions from *kRASG12D* alone expressing ERMS (S) and compared with those that co-express transgenic *ICN1* (T-U). Asterisk denotes a significant increase (p<0.01) in proliferation between *myf5-GFP+/mylz2-mCherry+* cells in *kRASG12D* and *kRASG12D+ ICN1* expressing tumors.

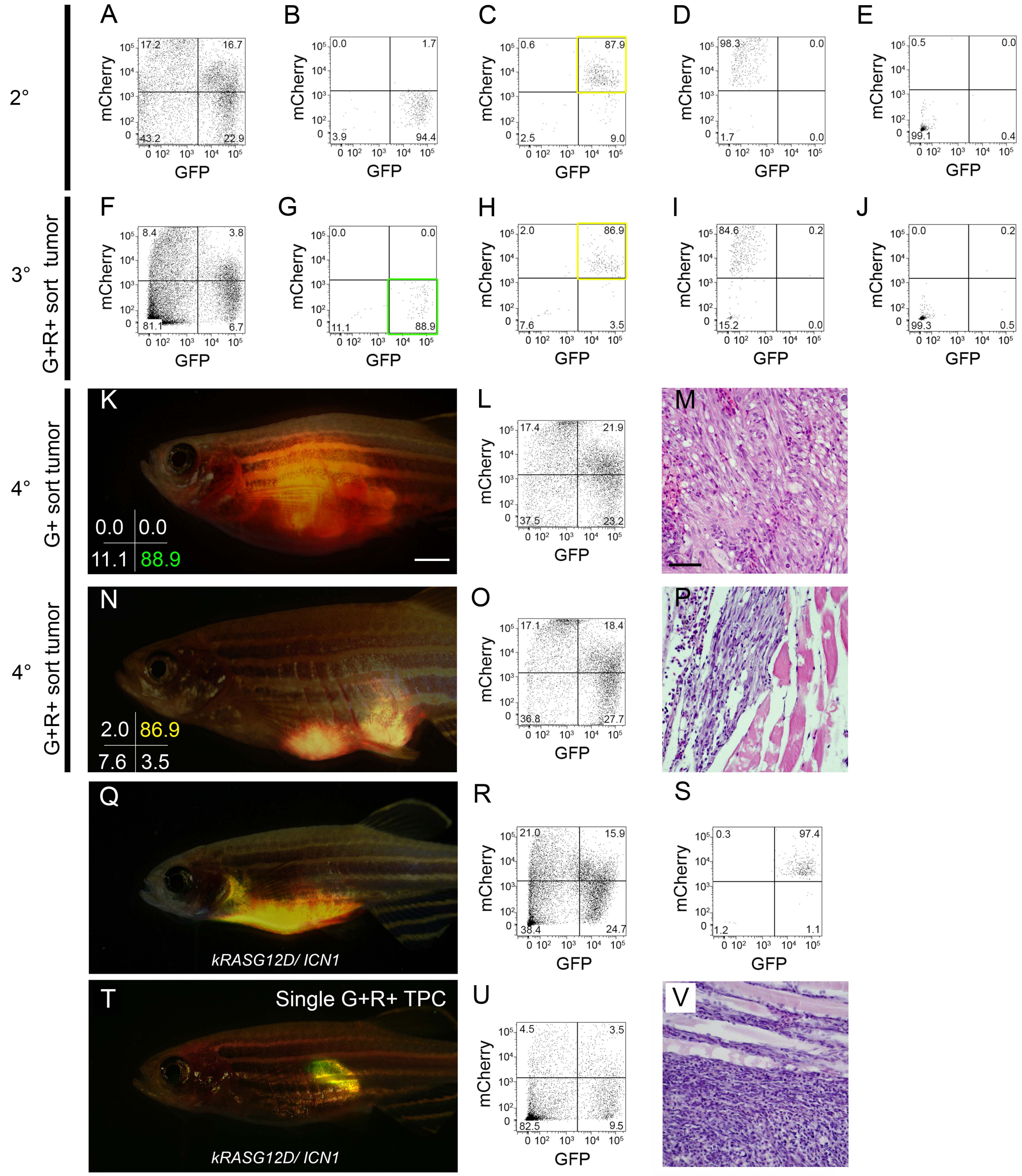

Figure S2. The double positive *myf5-GFP+/mylz2-mCherrry*+ ICN1-expressing ERMS cells generate tumors when engrafted into syngeneic CG1-strain recipient fish. Related to Figure 2.
(A-E) FACS plots of *kRASG12D* + *ICN1*-expressing ERMS (A) and following sorting of purified ERMS cell subfractions. These data show sort purity for experiments in Figure 2 B, E and Table S2. (F-J) FACS plots of tertiary transplanted ERMS (F) and following sorting of purified ERMS cell subfractions (G-J). (K-P) Representative animals serially transplanted with FACS sorted *myf5-GFP+/mylz2-mCherry-negative ICN1*-expressing ERMS cells (K-M, 1x10^3 cells engrafted/fish) or *myf5-GFP+/mylz2-mCherrry*+ cells (N-P, 1x10^3 cells engrafted/fish). Whole animal fluorescent images of engrafted fish with sort purity of transplanted tumor cells denoted in the lower left panel (K, N). Analysis of engrafted cell subfractions by FACs (L, O) or hematoxylin and eosin stained sections (M, P). (Q-V) Engraftment of highly purified double-positive *myf5-GFP+/mylz2-mCherry*+ ERMS cells verified acquisition of self-renewal and the ability to de-differentiate into *myf5-GFP*+ alone expressing cells. *myf5-GFP+/mylz2-mCherry*+ ERMS cells were isolated from transplanted fish and enriched following three rounds of FACS (Q-S). Single TPC equivalents were injected into CG1 recipient fish (T). Engrafted tumors were analyzed by FACS (U) and histology (V). Scale bar in K, N, Q and T equals 2mm; M, P, and V equals 50µm.

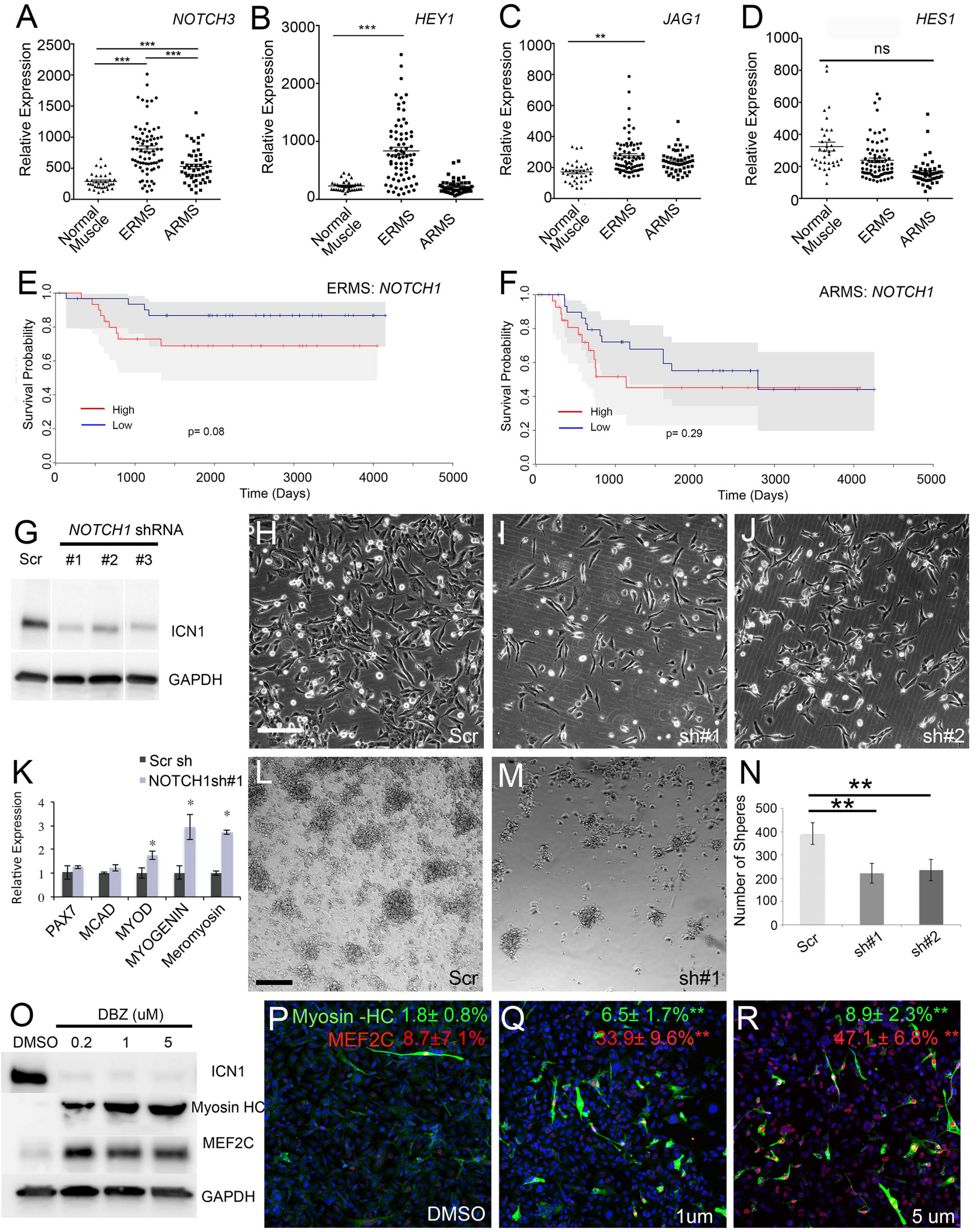

Figure S3. ***NOTCH1* is active in a large fraction of human RMS and regulates cell growth, differentiation and self-renewal in human SMS-CTR ERMS cells. Related to Figure 3.**
(A-D) Microarray gene expression analysis using the Triche data set of putative *NOTCH1* downstream target genes including *NOTCH3* (A), *HEY1* (B), *JAGGED1 (JAG1)* (C), or *HES1* (D). Analysis was completed using normal human skeletal muscle, ARMS, and ERMS. (E, F) Kaplan-Meijer analysis comparing survival in high vs. low *NOTCH1* expressing ERMS (p=0.08) and ARMS (p=0.29, Log-Rank statistic). (G) Western blot analysis of SMS-CTR cells following control shRNA (Scr) or *NOTCH1* knockdown using three independent lentiviral shRNA hairpins. (H-J) Morphology of SMS-CTR cells after 5 days of shRNA treatment. Control Scramble (H, Scr) and *NOTCH1* knockdown for shRNA#1 and #2, respectively (I, J), (K) Quantitative real-time PCR gene expression for a panel of muscle differentiation genes in control (Scr) and *NOTCH1* knockdown SMS-CTR cells. (L-N) Sphere formation in stable SMS-CTR knock-down cells. Images of spheres from scramble (L) or *NOTCH1* shRNA knockdown cells (M). Quantitation of sphere colony formation following seeding of $2x10^4$ cells/well (N). (O) Western blot of data depicted in Figure 3S bottom but includes expression of MEF2C. (P-R) Immunofluorescence staining for Myosin Heavy Chain (HC, green) and MEF2C (red) in RD cells treated with DMSO (P) or 1µm (Q) or 5µm (R) DBZ for 10 days and then grown under differentiation conditions. Percentage of tumor cells with Myosin HC expression are denoted +/- 1 standard deviation. ***, p<0.001; **, p<0.01; and * p<0.05 by Student's T-test. Error bars are +/- 1 STD. Scale bars equal 200µm in H-J, 400µm in L, M and 50 µm in P-R.

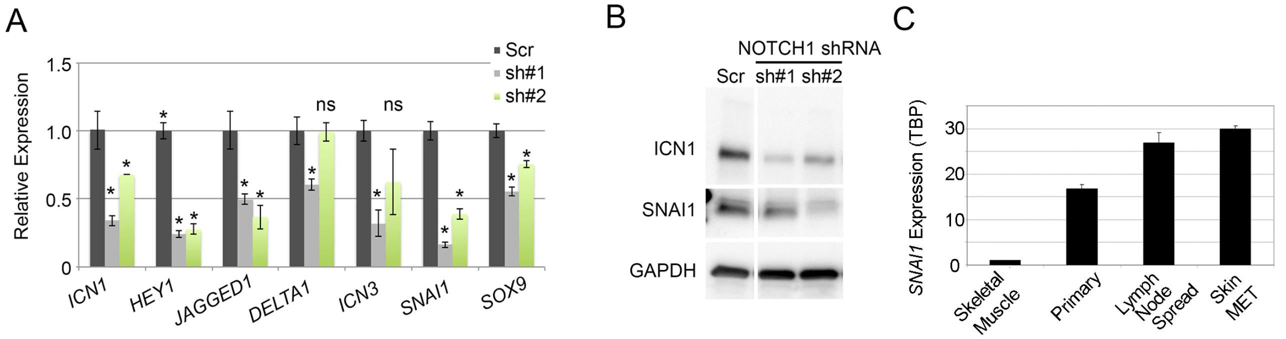

D

	NOTCH1	SNAI1	SNAI2	MEF2C	AXIN2	CCND1	DKK1	GLI1	GLI3	PTCH1	PTCH2	
NOTCH1		0.6	0.37	-0.1	0.16	0.21	-0.01	0.33	0.57	0.1	0.16	NOTCH1
SNAI1	0.0001		0.62	0.13	0.16	0.16	0.02	0.37	0.58	-0.04	-0.01	SNAI1
SNAI2	0.0001	0.0001		-0.02	0.18	0.51	0.14	0.54	0.72	0.05	0.03	SNAI2
MEF2C	0.2473	0.1368	0.8291		0.15	-0.22	0.07	-0.05	0.04	-0.08	-0.1	MEF2C
AXIN2	0.0715	0.0679	0.0398	0.0804		-0.02	0.21	0.2	0.39	0.23	0.27	AXIN2
CCND1	0.0149	0.0697	0.0001	0.0123	0.8224		0.29	0.43	0.39	0.16	0.07	CCND1
DKK1	0.9207	0.8501	0.1025	0.4482	0.0158	0.001		-0.02	0.13	0.21	0.15	DKK1
GLI1	0.0002	0.0001	0.0001	0.5448	0.0248	0.0001	0.831		0.61	0.29	0.32	GLI1
GLI3	0.0001	0.0001	0.0001	0.6298	0.0001	0.0001	0.1457	0.0001		0.14	0.19	GLI3
PTCH1	0.2686	0.6592	0.6098	0.3539	0.008	0.0775	0.0195	0.0008	0.1014		0.26	PTCH1
PTCH2	0.0686	0.9163	0.6982	0.2465	0.002	0.4029	0.0808	0.0003	0.0294	0.0024		PTCH2

E

	NOTCH1	SNAI1	SNAI2	MEF2C	NANOG	POU5F1	SOX2	
NOTCH1		0.6	0.37	-0.1	-0.12	-0.03	-0.06	NOTCH1
SNAI1	0.0001		0.62	0.13	-0.24	-0.18	-0.3	SNAI1
SNAI2	0.0001	0.0001		-0.02	-0.17	-0.04	-0.45	SNAI2
MEF2C	0.2473	0.1368	0.8291		-0.25	-0.34	-0.14	MEF2C
NANOG	0.1867	0.0055	0.0515	0.0042		0.39	0.3	NANOG
POU5F1	0.7378	0.0371	0.6137	0.0001	0.0001		0.18	POU5F1
SOX2	0.5203	0.0005	0.0001	0.1151	0.0005	0.0404		SOX2

Pearson Correlation
- ≥0.30
- 0.01 to 0.29
- NS
- -0.01 to -0.29
- ≤ -0.03
- P values

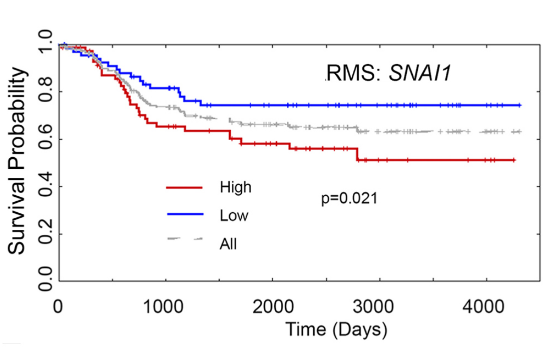

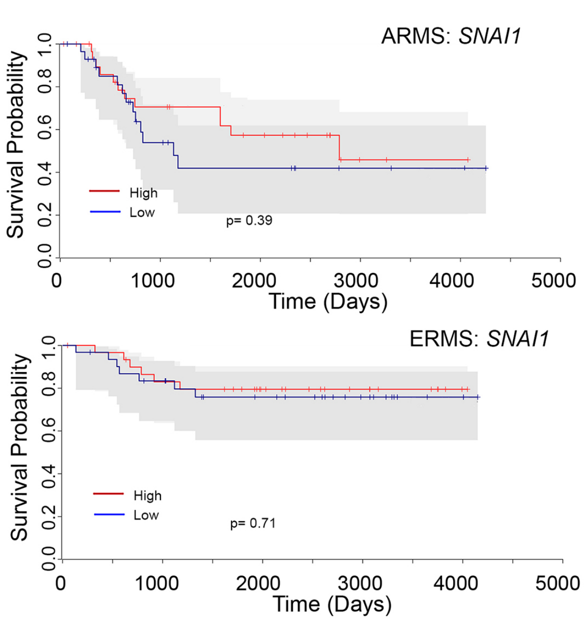

Figure S4. *SNAI1* is a downstream target of NOTCH1 in human RMS. Related to Figure 4.
(A) Quantitative real-time PCR gene expression of stable shRNA control (Scr) and NOTCH1 knockdown with two shRNA in RD cells. Single asterisk denotes p<0.01, Student's T-test. (B) Western blot analysis of human SMS-CTR cells following stable knockdown of NOTCH1. (C) An ARMS tumor with higher *SNAI1* expression in metastatic tumor growths when compared to primary tumor. (D) Pearson correlation between the expression of *NOTCH1, SNAI1, MEF2C, AXIN2, CCND1, DKK1, GLI1, GLI3, PTCH1* and *PTCH2* in primary human RMS assessed by RNA-sequencing analysis. (E) Pearson correlation between the expression of *NOTCH1, SNAI1, MEF2C, NANOG, POU5F1* and *SOX2* in primary human ERMS assessed by RNA-sequencing analysis. (F, H) Kaplan-Meijer analysis comparing survival in high vs. low *SNAI* expression in RMS patients (p=0.021, Log-Rank statistic n=128, Davicioni et al., 2010), or within ARMS or ERMS subtypes (p=0.39, p=0.71, respectively). Error bars are +/- 1 STD, * p <0.05 by Student's T-test in A and C.

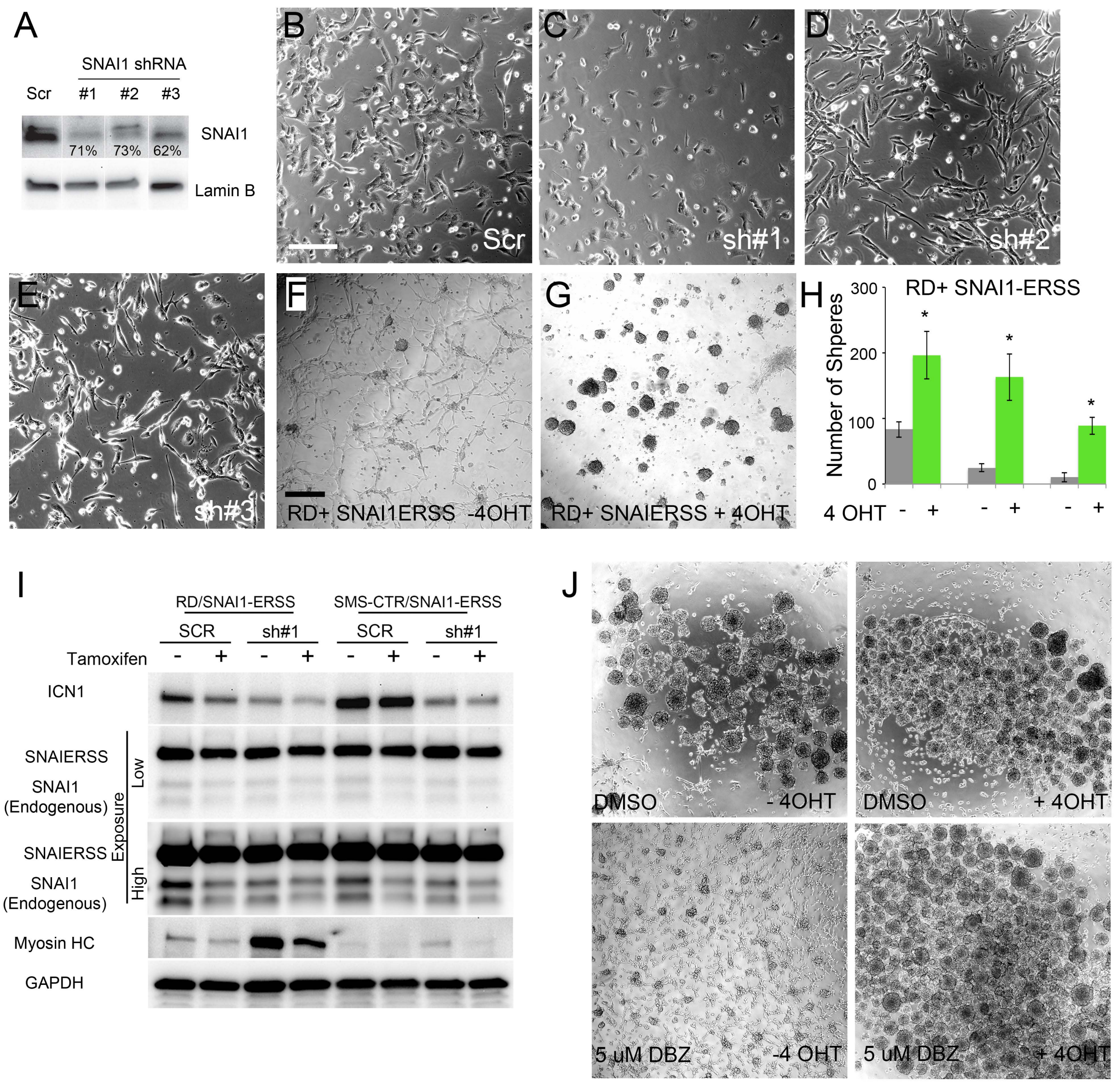

Figure S5. *SNAI1* regulates cell growth, self-renewal, and differentiation in human ERMS. Related to Figure 5. (A) Western blot analysis of SMS-CTR cells following control shRNA (Scr) or *SNAI1* knockdown using three independent lentiviral shRNA hairpins. Percent knockdown noted. (B-E) Morphology of SMS-CTR cells after 5 days of shRNA treatment. Control Scramble (B, Scr) and *SNAI1* knockdown (C-E). (F-H) Sphere formation in RD cells stably expressing SNAI1-ERSS with and without 4-hydroxytamoxifen treatment, (F and G, respectively). $1x10^4$ cells seeded per well, n=6/treatment. Quantitation showing elevated sphere colony formation following activation of SNAI1-ERSS in three independent experiments (H). Asterisks denote $p<0.05$. (I) Western blot analysis of RD and SMS-CTR cells that stably express SNAI-ERSS and have scramble control shRNA or ICN1 knockdown (sh#1). Cells were treated for 10 days with tamoxifen and then assessed by Western blot analysis. (J) Sphere formation in RD cells expressing SNAI1-ERSS and treated for 10 days with DMSO, Top left panel DMSO control image same image as in Figure 5Q, 5 μM DBZ and/or 1 μM tamoxifen as noted Figure 5T. A single data panel, DMSO -4OHT is reproduced from 5Q.

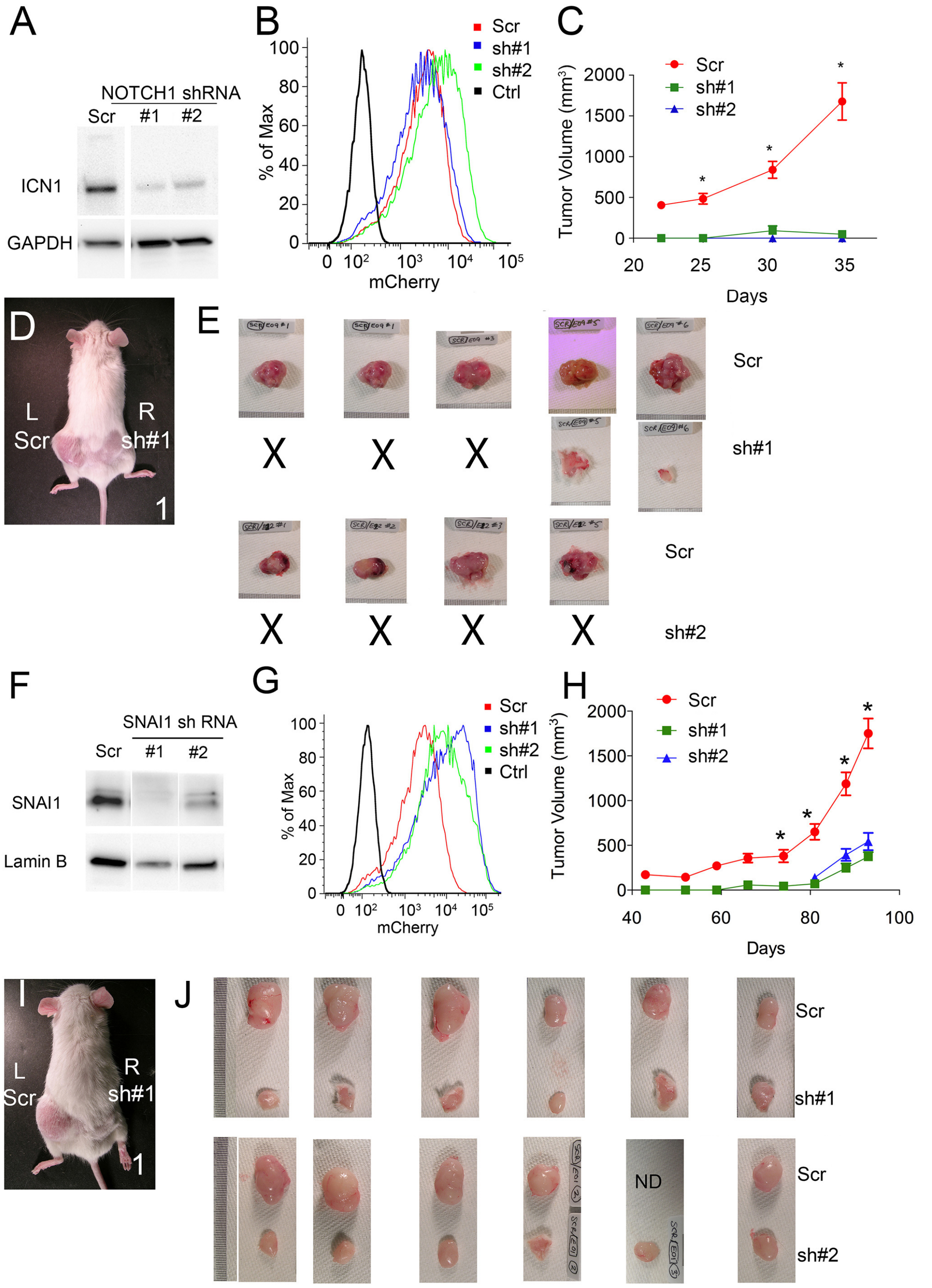

Figure S6. *NOTCH1* and *SNAI1* are required for growth and maintenance of human ERMS following xenograft transplantation into NOD/SCID/Il2gr-null mice. Related to Figure 6. (A-E) *NOTCH1* knockdown suppresses RD growth in xenograft transplanted mice. (A) Western blot analysis of RD cells following 3 days of infection with shRNA control or shRNAs to *NOTCH1*. (B) FACS analysis showing high transfection rates of cells. mKATE is co-expressed with luciferase in these experiments. (C) Tumor growth assessed by caliper measure between 25 and 36 days post-transplantation. Error bars denote +/-1 S.E.M and asterisks p<0.05, Student's T-test. (D) Representative image of a mouse engrafted with RD cells expressing scramble control (Scr, left flank) or NOTCH1 shRNA #1 (sh#1, right flank). (E) Images of excised tumors at necropsy. X denotes no tumor was found at necropsy. (F-J) *SNAI1* knockdown suppresses RD growth in xenograft transplanted mice. (F) Western blot analysis of RD cells following 3 days of infection with shRNA control or shRNAs to *SNAI1*. (G) FACS analysis showing high transfection rates of cells. mKATE is co-expressed with luciferase in these experiments. (H) Tumor growth assessed by caliper measure between 40 and 90+ days post-transplantation. Error bars denote +/-1 S.E.M and asterisks p<0.05, Student's T-test. (I) Representative image of mouse engrafted with RD cells expressing scramble control (scr, left flank), *SNAI1* shRNA #1 (sh#1, right flank). (J) Bottom, Images of excised tumors at necropsy. ND, not determined

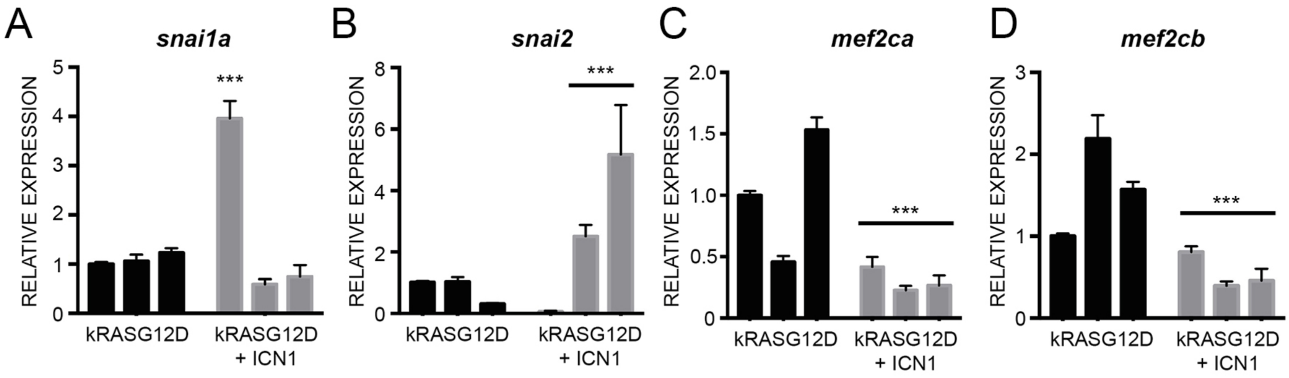

Figure S7. Quantitative real-time PCR analysis of the *snail/mef2c* axis in zebrafish *kRASG12D* and *kRASG12D+ICN1* expressing ERMS. Related to Figure 7.
Genes analyzed are denoted within each figure panel. ***, p <0.001 (Student's T-test, three independent tumors are shown for each genotype). In the tumors analyzed kRASG12D+ ICN1 expressing tumors have either high *snai1a* or *snai2*.

Table S1 related to Figure 1; Bulk tumor limiting dilution cell transplantation analysis comparing *kRASG12D (RAS) to kRASG12D + ICN1 (RAS+ICN1*) ERMS tumors

Tumor	Number of bulk tumor cells transplanted				TPC #	95% CI
	10000	1000	100	10		
RAS #1	0 of 6	1 of 7	1 of 9	0 of 6	1 in 33704	7770-147552
RAS #2	ND	0 of 4	0 of 4	0 of 5	1 in > 1485	1485-inf
RAS #3	ND	0 of 5	0 of 8	0 of 8	1 in >1963	1963-inf
RAS #4	ND	4 of 5	1 of 9	0 of 9	1 in 695	278-1742
				**Cumulative**	**1 in 3078**	**1454-6518**
RAS+ICN1 #1	6 of 6	2 of 5	1 of 8	0 of 9	1 in 1546	521-4587
RAS+ICN1 #2	ND	6 of 6	4 of 10	0 of 9	1 in 207	88-484
RAS+ICN1 #3	6 of 6	4 of 6	3 of 7	0 of 10	1 in 586	247-1391
RAS+ICN1 #4	6 of 6	4 of 5	3 of 7	2 of 7	1 in 293	113-761
				**Cumulative**	**1 in 499***	**323-771**

Asterisk denotes $p=1.44x10^{-6}$ for combined analysis using the Extreme Limiting Dilution Analysis software package.

Table S2 related to Figure 1 and 2; NOTCH1 confers TPC potential to mid-differentiated ERMS cells

Tumor	ERMS population	Number of sorted cells transplanted			TPC #	95% CI	Purity %
		1000	100	10			
RAS #1	G+	4 of 4	5 of 7	0 of 10	1 in 102	42-245	92.2
	**G+R+**	**0 of 5**	**0 of 9**	**0 of 9**	**1 in > 2000**	**2000-inf**	**86.6**
	R+	0 of 5	nd	nd		1669-inf	94.7
	Neg	0 of 5	nd	nd		1669-inf	99.9
RAS #2	G+	4 of 5	3 of 9	0 of 9	1 in 450	187-1082	95.7
	**G+R+**	**2 of 6**	**1 of 9**	**0 of 9**	**1 in 1952**	**611-6232**	**91.7**
	R+	0 of 5	nd	nd		1669-inf	96.7
	Neg	0 of 5	nd	nd		1669-inf	99.9
Serial Trasplant	G+	4 of 6	5 of 8	0 of 8	1 in 435	186-1014	92.1
	**G+R+**	**1 of 5**	**0 of 8**	**0 of 8**	**1 in 5364**	**736-37695**	**88.4**
RAS+ICN1 #1	G+	5 of 5	8 of 10	2 of 9	1 in 50	25-100	91.4
	**G+R+**	**3 of 5**	**3 of 10**	**0 of 10**	**1 in 682***	**277-1679**	**86.9**
	R+	0 of 5	nd	nd		1669-inf	98.5
	Neg	0 of 5	0 of 10	0 of 10		2036-inf	99.6
RAS+ICN1 #2	G+	5 of 6	3 of 7	0 of 9	1in 398	167-947	90.6
	**G+R+**	**4 of 4**	**3 of 9**	**1 of 10**	**1 in 164****	**66-405**	**91.9**
	R+	0 of 6	nd	nd		2003-inf	83.5
	Neg	0 of 4	nd	nd		1335-inf	99.3
RAS+ICN1 #3	G+	5 of 5	6 of 9	1 of 8	1 in 89	41-193	94.4
	**G+R+**	**7 of 7**	**4 of 8**	**0 of 9**	**1 in 164****	**66-405**	**87.9**
	R+	0 of 7	0 of 10	0 of 9		2701-inf	97.6
	Neg	0 of 7	0 of 10			2670-inf	93.2
Serial Trasplant	G+	6 of 7	na	na	1 in 514	202-1306	88.9
	**G+R+**	**6 of 6**	**3 of 10**	**1 of 3**	**1 in 204**	**85-490**	**88.4**
RAS+ICN1 #4	G+	5 of 6	7 of 8	2 of 8	1 in 165	66-412	96.4
	**G+R+**	**6 of 7**	**3 of 8**	**0 of 9**	**1 in 400****	**178-896**	**93.1**
	R+	0 of 5	0 of 7	nd		1903-inf	98.7
	Neg	0 of 5				1669-inf	99.5
Serial Trasplant	G+	4 of 4	6 of 7	2 of 8	1 in 46	20-104	88.8
	**G+R+**	**6 of 6**	**4 of 6**	**0 of 8**	**1 in 112**	**43-298**	**87.3**
RAS+ICN1 #5	G+	7 of 7	6 of 10	3 of 10	1 in 80	39-165	88.9
	**G+R+**	**7 of 7**	**5 of 9**	**2 of 10**	**1 in 100****	**46-219**	**90.7**
	R+	0 of 7	nd	nd		2337-inf	97.1
	Neg	0 of 7	nd	nd		2337-inf	98
Serial Trasplant	G+	5 of 6	5 of 9	0 of 9	1 in 306	131-1770	87.2
	**G+R+**	**4 of 6**	**2 of 9**	**0 of 9**	**1 in 742**	**311-1770**	**91.4**

Single asterisk denotes p=0.011, Extreme Limiting Dilution Analysis

Two asterisks denotes p<0.0005, Extreme Limiting Dilution Analysis

Table S3 related to Figure 2; Mid-differentiated ERMS cells can transplant tumor

Tumor	ERMS Cell Subpopulation	TPC frequency	95% CI	Purity	Animals Engrafted	Probability of engraftment from FACs sorted population
RAS+ICN #3<br>(20 cells)	G+<br>G+R+	1 in 89<br>1 in 164	41-193<br>66-405	97.2 %<br>97.4%	1 in 10<br>2 in 20	99.71%<br>99.85%
RAS+ICN1 #4<br>(10 cells)	G+<br>G+R+	1 in 165<br>1 in 400	66-412<br>178-896	90.5%<br>>84%	3 in 10<br>1 in 20	99.74%<br>99.82%

Table S4; De-identified human RMS tumors for Q-PCR analysis. Related to Figure 3B, 4C and S4C.

	Label in Graph	Stage	Histology	Fusion status
SM				
RD				
RH6				
RH18				
	ERMS			
R4113-04	1	1	ERMS	
S08-2301A	2	3	ERMS	
S06-3846A	3	3	ERMS	
R4107-04	4	ND	ERMS	
S07-1138C	5	3	ERMS	
	ARMS			
R4147-03	1	4	ARMS	t negative
R4148-03	2	4	ARMS	t negative
R4014-04	3	3	ARMS	t(2;13)
S07-734A	4	3	ARMS	t(1;13)
S05-1895A	5	3	ARMS	t(2;13)
R4011-04	6	3	ARMS	t(2;13)
R4121-03	7	4	ARMS	t(2;13)

Table S5; Primers

Human					
**Gene**	**Primer**	**Sequence**	**Gene**	**Primer**	**Sequence**
DELTA1	Forward	CTACTACGGAGAGGGCTGCT	*c-SNAI2*	Forward	GAACTGGACACACATACAGTGATT
	Reverse	CCAGGGTTGCACACTTTCTC		Reverse	GAGAGAGGCCATTGGGTAGC
GAPDH	Forward	GGTGGTCTCCTCTGACTTCAACA	*SNAI2*	Forward	CAGACCCTGGTTGCTTCAA
	Reverse	GTTGCTGTAGCCAAATTCGTTGT		Reverse	TGACCTGTCTGCAAATGCTC
c-HES1	Forward	CAGCCAGTGTCAACACGACA	*SOX9*	Forward	GACGCTGGGCAAGCTCT
	Reverse	ATGCCGCGAGCTATCTTTCTT		Reverse	GTAATCCGGGTGGTCCTTCT
HES1	Forward	TCAACACGACACCGGATAAA			
	Reverse	CCGCGAGCTATCTTTCTTCA	c-RPS18-F&R	F,R	BioRad qHsaCED0037454
c-HEY1	Forward	GGTAATGGAGCAAGGATCTGCTA	c-TBP-F&R	F,R	BioRad qHsaCID0007122
	Reverse	CCTTTCCCTCCTGCCGTATG	c-RPLP0-F&R	F,R	BioRad qHsaCED0038653
HEY1	Forward	CGAGGTGGAGAAGGAGAGTG			
	Reverse	CTGGGTACCAGCCTTCTCAG	**ChIP Snai1**		
JAGGED1	Forward	GACTCATCAGCCGTGTCTCA	-940 bp	Forward	GCCCTTGAGGCAAAGTCCAA
	Reverse	TGGGGAACACTCACACTCAA	-940 bp	Reverse	GTCGGGGTGACttcccagag
JDP2	Forward	TGGAGGAGCTGAAATACGCT	-843 bp	Forward	CCATCCCTGGAAGCTGCTCT
	Reverse	CCTTTTCCTTCGCTCCTCTT	-843 bp	Reverse	GTGCTTTGGTGGGGGAGAGA
MCADHERIN	Forward	GGAGGACCAGGACGCCTACGA	-420 bp	Forward	CGCTTCCTCCCCAGTGATGT
	Reverse	AGGCTGTCCGGCCTCTGTGT	-420 bp	Reverse	CGGTGGTCTGAGCGCTTCT
1 MEF2C	Forward	CCAAGGACTAATCTGATCGGG	+7.5kb(-ve ctl)	Forward	GAGCCCTGGAAGAGCTGAAT
	Reverse	TTTCCTGTTTCCTCCAAACAA	+7.5kb(-ve ctl)	Reverse	ACTCTCCTCTCCCACCCTGT
2 MEF2C	Forward	TTCAACGCTGGACGAAGTAA	NG RegionCh6	Forward	GCCTGGGATGGAAACCATGT
	Reverse	AATTCCTGCATTCGTTCCTG	NG RegionCh6	Reverse	CCACCAGCACCAGAAGGAGA
3 MEF2C	Forward	TTCAACGCTGGACGAAGTAA			
	Reverse	CAGTTCCCAAATTCCTGCAT	**Zebrafish**		
MYOD1	Forward	CGCGACGTAGACCTGACGGC	*dusp4*	Forward	ACGAACTTTGCGAAATGGAC
	Reverse	GTGGTCTTGCGCTTGCACGC		Reverse	CTGCAATCCAGAAGCAGACA
MYOGENIN	Forward	CCTGCCGTGGGCGTGTAAGG	*dusp6*	Forward	ATTGCGGGAAAATCAGTTTG
	Reverse	GGACTGCAGGAGGCGCTGTG		Reverse	CTGGAGCCAGAACCTCTCAC
MEROMYOSIN	Forward	GTGAGAGGCTGGAAGAAGCCGTG	mef2ca 21F	Forward	GATTGCGCGGATAATGGACG
	Reverse	TCCTCCAGGTCCCTGCGCATT		Reverse	AATACGCTCAGCTCGTAGGC
c-NOTCH1	Forward	GCAGAGGCGTGGCAGACTAT	mef2cb 78F	Forward	TGGCCTGATGAAGAAAGCCT
	Reverse	GCTGGCACGATTTCCCTGAC		Reverse	CTGGAACAGCTTGTTGGTGC
NOTCH1	Forward	CACTGTGGGCGGGTCC	*myh9a*	Forward	GAGGGGTCCGAGCCAACTCC
	Reverse	GTTGTATTGGTTCGGCACCAT		Reverse	AGAGCATGATGGCTGATAGCAGGT
c-NOTCH3	Forward	CCTGTGGCCCTCATGGTATC	*notch1a*	Forward	CGACGGTGGCACAAGGGCAA
	Reverse	CATGGGTTGGGGTCACAGTC		Reverse	TGGGTTGCAGGGGTTCGGGA
NOTCH3	Forward	TGGCGACCTCACTTACGACT	*notch3*	Forward	GCTCCTGCCCTCCAGGAACAC
	Reverse	CACTGGCAGTTATAGGTGTTGAC		Reverse	TCCCCGAGGCCATGAGGGAG
PAX7	Forward	TGTGACCGAAGCACTGTGCCC	*snai1a*	Forward	CATACAGGTGAGCGTCCGTT
	Reverse	AGCCGGTTCCCTTTGTCGCC		Reverse	CTTCACATCTGCGTGGGTCT
RUNX1	Forward	CCACTCCACTGCCTTTAACC	*snai1b*	Forward	CGCTGAAGTTTCGAGGGGAT
	Reverse	CTGGGTGCACAGAAGGAGAG		Reverse	TGCATATCGCCGGTCAGTC
c-SNAI1	Forward	TAGCGAGTGGTTCTTCTGCG	*snai2*	Forward	ACTTCAGCATGCCTCGTTCA
	Reverse	CTGCTGGAAGGTAAACTCTGGAT		Reverse	ATAAACACTGTTGGACTCTCCAGT
SNAI1	Forward	CTTCCAGCAGCCCTACGAC			
	Reverse	CGGTGGGGTTGAGGATCT			

* c- primers used for real time Q-PCRexperiments in Primary RMS
* NG Region corresponds to Non Genic region on a different chromosome

Supplemental Methods and Materials

Animals

Zebrafish used in this work include: CG1 strain (Mizgireuv and Revskoy, 2006), *myf5*-GFP transgenic zebrafish, *myf5-GFP/mylz2-mCherry* double transgenic fish CG1-strain zebrafish syngeneic transgenic zebrafish (Ignatius et al., 2012). 8-week-old female NOD/SCID/IL2g-null mice used in this study and were obtained from Jackson laboratory, Maine Bar Harbor.

Micro-injection and ERMS generation

rag2-kRASG12D, rag2-ICN1, rag2-GFP, rag2-dsREDexpress constructs were described previously (Blackburn et al., 2012; Langenau et al., 2007; Smith et al., 2010). The constructs were linearized with *Xho1*, phenol:chloroform-extracted, ethanol-precipitated, resuspended in 0.5× Tris-EDTA + 0.1 M KCl, and injected into one-cell stage embryos of the respective backgrounds, as previously described (Langenau et al., 2007).

Quantification of zebrafish RMS size and initiation

Zebrafish were followed for time to tumor onset using an epifluorescent stereomicroscope. Primary tumor size was quantified at 30 days of age using fluorescence intensity multiplied by the pixel area using the ImageJ software package as described previously (Chen et al., 2014). Kaplan-Meier tumor onset analysis was performed using Graphpad Prism® Software.

Histology, immunohistochemistry, Edu staining

Paraffin embedding, sectioning and immunohistochemical analysis of zebrafish and mouse tumor sections were performed as described (Chen et al., 2014; Ignatius et al. 2012). Antibodies used for immunohistochemistry included: NOTCH1 (1:1000, D1E11) XP rabbit mAB (Cell Signaling) and KI67. All histopathology procedures were performed at the MGH and BWH DF/HCC Research Pathology Cores. Slides were imaged using a transmitted light Olympus BX41 microscope. Pathology review and staging were completed by two board-certified sarcoma pathologists (G.P.N and E.Y.C).

Edu was injected into the peritoneum of ERMS burdened zebrafish (10 mM Edu in 5 µl PBS containing 5% FBS). 6 h post injection the fish were euthanized and fixed in 4% PFA at 4 °C overnight and then embedded in OCT. Frozen blocks were sectioned at 8-10 micron thickness and stained for Edu (Click iT Alexa Fluor 647 imaging kit, Invitrogen). Sections were mounted in Vectashield with dapi, and then imaged on a confocal microscope.

FACS and ERMS cell transplantation

FACS analysis and RMS cell transplantation were completed essentially as described (Chen et al.,2014; Ignatius et al., 2012; Langenau et al., 2007). Zebrafish ERMS tumor cells were stained with DAPI to exclude dead cells and sorted twice using a Laser BD FACS Aria II Cell Sorter. Sort purity and viability were assessed after two rounds of sorting when possible, exceeding 85% and 95% respectively. ERMS tumors that were fluorescently labeled with GFP, dsRED or mCherry were transplanted into syngeneic CG1 Fish and were monitored for tumor engraftment under a fluorescent dissecting microscope from 10 to 120 days post transplantation. Tumor-propagating cell frequency was quantified following transplantation into CG1 syngeneic recipient fish using the Extreme Limiting Dilution Analysis software (http://bioinf.wehi.edu.au/software/elda/). Subpopulations or ERMS total tumor from a subset of transplanted fish were sorted into tubes and fixed with RLT buffer for RNA isolation which was used to generate cDNA for quantitative realtime PCR experiments. Subsets of tumors were fixed in 4% PFA, sectioned, stained with Hematoxylin and Eosin.

Bioinformatic analysis of human RMS samples

Previously published transcriptome data from 65 ERMS samples (Shern et al., 2014) were processed using a standard Tuxedo pipeline (Trapnell et al., 2012). The resulting gene expression from transcriptome datasets were then log2 transformed and standardized (z-scored) using a set of 63 normal tissue samples. Using “Hmisc” package in R, Pearson correlation was determined between following genes: NOTCH1, *NOTCH3, SNAI1, SNAI2, MEF2C, HES1, HEY1, GLI1, GLI3, PCTH1, PTCH2, AXIN2, CCND1, DKK1, NANOG, POU5F1 and SOX2* using the standardized data.

Western blot analysis

Total cell lysates from human RMS cell lines and human myoblasts were obtained following lysis in 2%SDS lysis buffer supplemented with protease inhibitors (Santa Cruz Biotechnology). Samples were boiled, vortexed and homogenized through a syringe. 40 μg of protein was loaded in 4-20% Mini-Protean TGX gels (Biorad) and transferred onto PVDF membranes. Western blot analysis used primary antibodies: rabbit a-NOTCH1 (1:5000, Abcam ab125078), Cleaved NOTCH1 antibody (1:600-1000, Val1744, D3B8 rabbit mAB, Cell Signaling), a-SNAI1 goat pAB (1:400, R&D systems, AF3639), a-Myosin Heavy Chain mouse mAB MF20 (R&D systems and DSHB A4.1025), MEF2C (D80C1) XP rabbit mAB (1:1000, Cell Signaling) and secondary antibodies: HRP anti-rabbit (1:2000, Cell Signaling 7074) or HRP anti-mouse (1:3000, GE Healthcare NA93IV). Blocking was completed using 3% skim milk/TBST. Membranes were developed using a ECL reagent (Western Lightening Plus-ECL, Perkin Elmer or sensitive SuperSignal West femto Maximum Sensitivity Substrate, Thermo Scientific). Membranes were striped, rinsed and re-probed with the respective internal control rabbit a-Lamin B1 (1:2500, Abcam ab4074) or rabbit a-GAPDH (1:2000, Cell Signaling 1:2000).

ChIP assay

Chromatin from $5x10^6$ RD cells was isolated and fixed with 1% formaldehyde and sonicated. Samples were processed according to the manufacturer's protocols (ChIP kit, Millipore). Immunoprecipitation was performed using 5μg of rabbit anti-NOTCH1 antibody (Abcam, ab27526) or rabbit IgG and Protein A/G agarose (Thermo Scientific). The immunoprecipitated DNA was subjected to real-time PCR with primers that target the human SNAI1 promoter (predicted NOTCH1 binding sites within -1kb of transcription start site was determined using the Find Individual Motif Occurrences tool (FIMO) (Grant et al., 2011) and a negative control region 7.5kb upstream as well as an unrelated ORF free region in Chr 6. All signals were normalized against input by percentage input calculation method and normalized to IgG signal. Significance was calculated by Student's T-test.

Plasmids, retroviral, lentiviral and siRNA constructs

The shRNA plasmids for NOTCH1 and SNAI1 knockdown were obtained from the Massachusetts General Hospital – Molecular Profiling Core Facility. NOTCH1 shRNA sequences are as follows sh#1 CTTTGTTTCAGGTTCAGTATT, sh#2 CAAAGACATGACCAGTGGCTA and sh#3 CGCTGCCTGGACAAGATCAAT. SNAI1 shRNA sequences are sh#1 CCAAGGATCTCCAGGCTCGAA, sh#2 TACAGCTGCTTTGAGCTACAG, and sh#3 CCACTCAGATGTCAAGAAGTA. The FUdeltaGW-rtTA plasmid was a gift from Dr. Konrad Hochedlinger. (Addgene Plasmid #19780) (Maherali et al., 2008), the TetO-MEF2C plasmid a gift from Dr. John Gearhart, Addgene Plasmid #46031) (Addis et al., 2013); the TetO-FUW-MEF2C plasmid a gift from Dr. Rudolf Jaenisch (Addgene Plasmid #61538) (Cassady et al., 2014); the NOTCH1ΔE retroviral vector was a gift from Dr. Raphael Kopan (University of Washington). The SNAI1ERSS plasmid was a gift from Dr. Daniel Haber (Massachusetts General Hospital). SMARTpool ON-TARGETplus human MEF2C siRNA (Dharmacon, Inc. L-009455-00-0005) and ON-TARGETplus Non-targeting Pool siRNA's were purchased from Dharmacon (Dharmacon, Inc. D-001810-10-05).

Immunofluorescence Staining

Gene-specific smart-pool or control siRNAs (Dharmacon, GE Life Sciences) (1pmol) were reverse-transfected into cells using RNAiMax lipofectamine transfection reagent (Life Technologies) in flat clear bottom 96 well plates. Cells were then fixed at 72 hours post transfection in 4% PFA/PBS. Cells were washed in x1PBS, permeabilized in 0.5% TritonX-100/PBS, washed again, and then incubated with the respective primary antibodies: rabbit a-MEF2C (1:500, Cell Signaling) and a-MF20 (1:250) in 2% goat serum/PBS. Secondary antibodies detection used Alexa 488 goat anti-mouse (1:1000, Invitrogen A11029) and Alexa 594 goat anti-rabbit (1:1000 Invitrogen A11037). Cells were washed in 1x PBS, incubated with DAPI (1:10,000), and imaged using a LSM710 Zeiss Laser scanning confocal microscope. Images were processed in ImageJ and Adobe Photoshop.

Lentiviral, retroviral and si-RNA knock-down

Scramble control shRNA and gene-specific shRNAs were delivered on the pLKO.1-background vector and packaged using 293T cells. RMS cells were plated in 6-well or 10 cm^2 tissue culture-grade plates, infected with viral particles for 24h at 37°C with 4 μg/ml of polybrene (EMD Millipore). Cells were then maintained in

DMEM/10%FBS media (RD, SMS-CTR cells). Cell lysates were collected 72 hours after infection and assessed for knockdown by Western blot analysis.

Viral supernatents were prepared by transfecting Plat-A retroviral packaging cells with pBabe-SNAI1ERSS or NOTCH1ΔE-EGFP using FuGENE6 (Promega). Stable pools of ERMS cells were generated by retroviral infection and selected using puromycin selection for SNAI1ERSS or FACsorting of GFP-positive NOTCH1ΔE-EGFP expressing cells. Gene-specific smart-pool or control siRNAs (Dharmacon, GE Life Sciences) (1pmol) were reverse-transfected into cells using RNAiMax lipofectamine transfection reagent (Life Technologies) in flat clear bottom 96 well plates. Cells were then grown for 72 hours under differentiation conditions and then fixed at 72 hours post transfection in 4% PFA/PBS.

Mouse xenografts, bioluminescent imaging, caliper measurements

RD ERMS cells were co-infected with shRNA lentivirus as outlined above. At 3 days post-infection, cells were collected and counted. An aliquot of cells was analyzed by using the SORP4 Laser BD LSRII Flow Cytometer to determine viability following DAPI staining. Equal numbers of viable cells were then embedded into matrigel at a final concentration of $1x10^6$ of viable cells per 100 μl. Six to eight week old NOD/SCID/IL2g-null mice were anesthetized by isofluorane and injected with RD scramble-shRNA/mKate-luc cells subcutaneously into the left flank whereas RD sh-NOTCH1 or SNAI1/mKate-luc cells injected on the right (100 μl/flank injection). Tumor growth was monitored weekly using bioluminescence imaging following injection of mice subcutaneously with a 100 μl volume of Luciferin at 75 mg/kg (15mg/ml injected IP) for the first 30 days. Imaging was completed using the IVIS Lumina II (Caliper Life Science). Comparison between groups was performed using a Student's T-test. After 30 days or when palpable tumors were obtained, tumor growth was measured using a caliper scale to measure the greatest diameter and length, which were then used to calculate the volume of the tumor.

Supplemental Methods References

Addis, R. C., Ifkovits, J. L., Pinto, F., Kellam, L. D., Esteso, P., Rentschler, S., Christoforou, N., Epstein, J. A., and Gearhart, J. D. (2013). Optimization of direct fibroblast reprogramming to cardiomyocytes using calcium activity as a functional measure of success. J Mol Cell Cardiol 60, 97-106.
Blackburn, J. S., Liu, S., Raiser, D. M., Martinez, S. A., Feng, H., Meeker, N. D., Gentry, J., Neuberg, D., Look, A. T., Ramaswamy, S., et al. (2012). Notch signaling expands a pre-malignant pool of T-cell acute lymphoblastic leukemia clones without affecting leukemia-propagating cell frequency. Leukemia 26, 2069-2078.
Cassady, J. P., D'Alessio, A. C., Sarkar, S., Dani, V. S., Fan, Z. P., Ganz, K., Roessler, R., Sur, M., Young, R. A., and Jaenisch, R. (2014). Direct lineage conversion of adult mouse liver cells and B lymphocytes to neural stem cells. Stem Cell Reports 3, 948-956.
Davicioni, E., Anderson, J.R., Buckley, J.D., Meyer, W.H., Triche T.J. (2010). Gene Expression Profiling for Survival Prediction in Pediatric Rhabdomyosarcomas: A Report from the Children's Oncology Group. J Clin Oncol. 2010 March 1; 28(7):1240-1246
Grant, C. E., Bailey, T. L., and Noble, W. S. (2011). FIMO: scanning for occurrences of a given motif. Bioinformatics 27, 1017-1018.
Hu, Y, and Smyth, GK (2009). ELDA: Extreme limiting dilution analysis for comparing depleted and enriched populations in stem cell and other assays. Journal of Immunological Methods 347, 70-78.
Maherali, N., Ahfeldt, T., Rigamonti, A., Utikal, J., Cowan, C., and Hochedlinger, K. (2008). A high-efficiency system for the generation and study of human induced pluripotent stem cells. Cell Stem Cell 3, 340-345.
Mizgireuv, I. V., and Revskoy, S. Y. (2006). Transplantable tumor lines generated in clonal zebrafish. Cancer Res 66, 3120-3125.
Smith, A. C., Raimondi, A. R., Salthouse, C. D., Ignatius, M. S., Blackburn, J. S., Mizgirev, I. V., Storer, N. Y., de Jong, J. L., Chen, A. T., Zhou, Y., et al. (2010). High-throughput cell transplantation establishes that tumor-initiating cells are abundant in zebrafish T-cell acute lymphoblastic leukemia. Blood 115, 3296-3303.
